# Robust and Sustained STING Pathway Activation via Hydrogel-Based In Situ Vaccination for Cancer Immunotherapy

**DOI:** 10.1021/acsnano.3c12337

**Published:** 2024-10-15

**Authors:** Sheng-Liang Cheng, Hsin-Mei Lee, Chung-Pin Li, Mei-Wei Lin, Min-Yuan Chou, Yu-Ting Yen, Tun-Han Wu, Yun-Chen Lian, Yu-Chuan Shih, Chi-Shiun Chiang, Ting-Wen Chen, Dehui Wan, Yunching Chen

**Affiliations:** † Institute of Biomedical Engineering, National Tsing Hua University, Hsinchu 30013, Taiwan; ‡ International Intercollegiate Ph.D. Program, National Tsing Hua University, Hsinchu 30013, Taiwan; § Division of Gastroenterology and Hepatology, Department of Medicine, Taipei Veterans General Hospital, Taipei 11217, Taiwan; ∥ Division of Clinical Skills Training, Department of Medical Education, Taipei Veterans General Hospital, Taipei 11217, Taiwan; ⊥ Therapeutic and Research Center of Pancreatic Cancer, Veterans General Hospital, Taipei 11217, Taiwan; # School of Medicine, College of Medicine, National Yang Ming Chiao Tung University, Taipei 112304, Taiwan; ¶ Biomedical Technology and Device Research Laboratories, Industrial Technology Research Institute, Hsinchu 310401, Taiwan; ∇ Institute of Translational Medicine and New Drug Development, School of Medicine, China Medical University, Taichung 406040, Taiwan; ○ Department of Biomedical Engineering and Environmental Sciences, National Tsing Hua University, Hsinchu 30013, Taiwan; ⧫ Institute of Bioinformatics and Systems Biology, National Yang Ming Chiao Tung University, Hsinchu 30068, Taiwan; †† Department of Biological Science and Technology, National Yang Ming Chiao Tung University, Hsinchu 30068, Taiwan; ‡‡ Center for Intelligent Drug Systems and Smart Bio-devices, National Yang Ming Chiao Tung University, Hsinchu 30068, Taiwan; §§ Department of Chemistry, National Tsing Hua University, Hsinchu 30013, Taiwan

**Keywords:** STING agonist, silk fibroin hydrogel, immunogenic cell death, cGAMP, neoadjuvant immunotherapy, cancer immunotherapy, nanoparticle

## Abstract

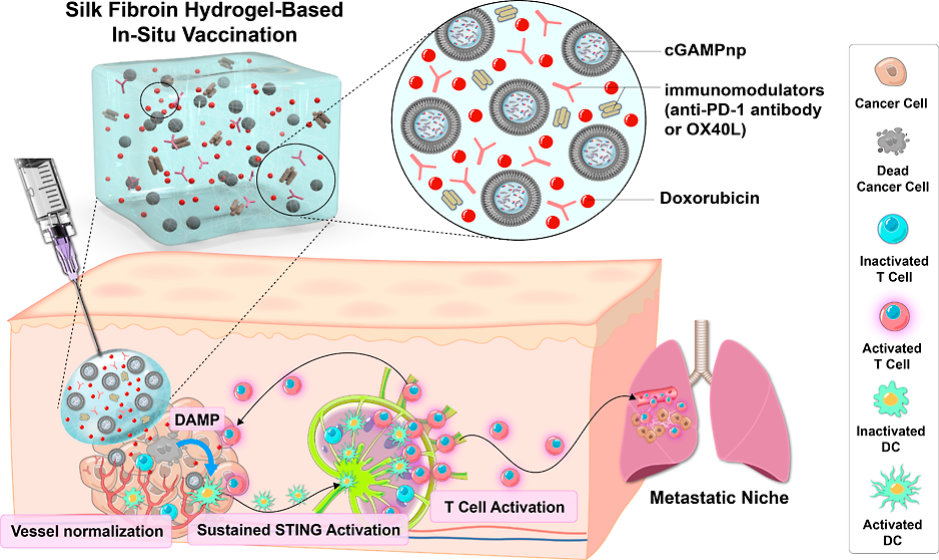

The stimulator of interferon genes (STING) pathway is crucial for tumor immunity, leading to the exploration of STING agonists as potential immunotherapy adjuvants. However, their clinical application faces obstacles including poor pharmacokinetics, transient activation, and an immunosuppressive tumor microenvironment (TME). Addressing these limitations, our study aims to develop an injectable silk fibroin hydrogel-based in situ vaccine. It incorporates a nanoscale STING agonist, an immunogenic cell death (ICD) inducer, and an immunomodulator to ensure their controlled and sustained release. cGAMP nanoparticles (cGAMPnps) with a core–shell structure ensure optimal delivery of cGAMP to dendritic cells (DCs), thereby activating the STING pathway and fostering DC maturation. ICD-associated damage-associated molecular patterns amplify and prolong STING activation via enhanced type I IFN and other inflammatory pathways, along with delayed degradation of cGAMP and STING. Furthermore, the STING-driven vascular normalization by cGAMPnps and ICD, in conjunction with immunomodulators like antiprogrammed cell death protein 1 antibody (anti-PD-1 Ab) or OX40 ligand (OX40L), effectively remodels the immunosuppressive TME. This in situ gel vaccine, when used independently or with surgery as neoadjuvant/adjuvant immunotherapy, enhances DC and CD8^+^ T-cell activation, suppressing tumor progression and recurrence across various immunologically cold tumor models. It revolutionizes the application of STING agonists in cancer immunotherapy, offering substantial promise for improving outcomes across a broad spectrum of malignancies.

## Introduction

Cancer immunotherapy has provided therapeutic options for patients with metastatic malignancies; for example, therapeutic cancer vaccines have been approved by the FDA for the treatment of various cancers.^1−4^ Notably, in situ cancer vaccines stand out, repurposing the tumor into an active vaccine-manufacturing site, targeting both the primary tumor and distant metastases, showing the innovation in cancer immunotherapy.^5−7^ The underlying mechanism of this approach consists of two critical components: a cytotoxic agent and an immune adjuvant. The cytotoxic agent facilitates antigen release and initiates immunogenic cell death (ICD) in the cancer cells.^8−12^ Moreover, a potent immune adjuvant is essential for enhancing intratumoral dendritic cell (DC) activation, strengthening both the innate and adaptive immune responses, and promoting long-term anticancer immunity.^13−17^

Stimulator of interferon genes (STING) agonists, including cyclic dinucleotides (CDNs), hold promise as adjuvants for cancer immunotherapy due to their capability to trigger the cyclic GMP–AMP synthase (cGAS)–STING pathway, thereby initiating tumor-specific adaptive immune responses.^18−24^ This pathway drives a comprehensive type I IFN response, promoting DC maturation and migration and priming cytotoxic T lymphocytes along with natural killer cells. Despite their potential demonstrated in preclinical studies, transitioning STING agonists to clinical use presents challenges.^25−27^ Notably, the hydrophilic properties of CDNs, coupled with their susceptibility to rapid enzymatic degradation, attenuate their bioavailability, leading to transient and moderate immune activation. While advanced STING-activating agents are being designed to counteract these limitations with improved bioavailability, they could present systemic side effects if they are not targeted to tumor sites. Thus, strategies are needed to address these challenges, such as effectively delivering cGAMP to the desired immune cells and mitigating its rapid degradation and adverse effects.^28,29^

**Figure 1:**
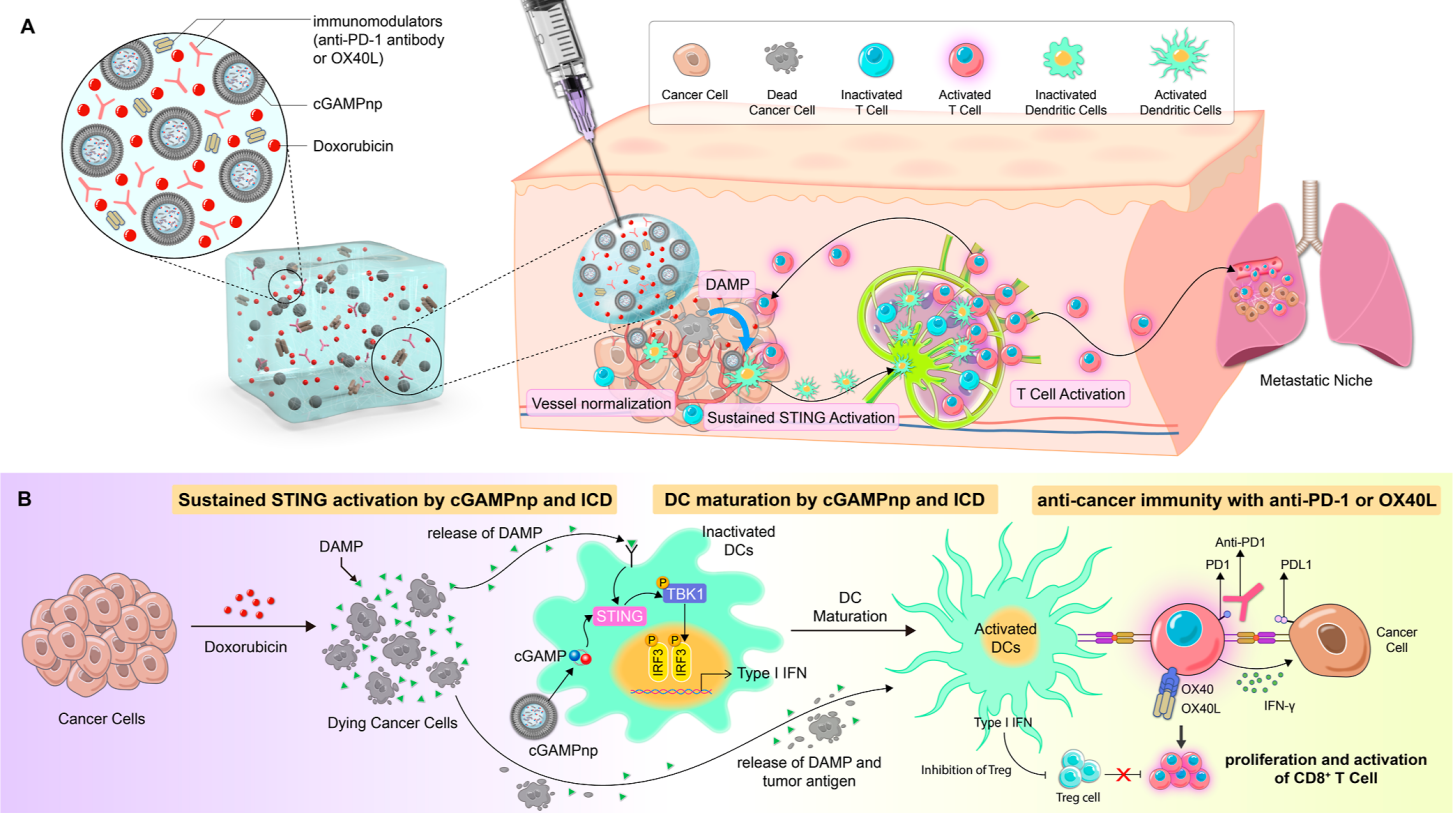
Schematic representation of in situ gel vaccination for the sustained release of cGAMPnps, Dox, and an immunomodulator to generate durable STING activation and effective anticancer immunity. (A) Thermoresponsive SF hydrogel-based in situ gel vaccine encapsulates a nanoscale STING agonist (cGAMPnp), an ICD inducer, and an immunomodulator, providing a localized depot for the controlled, sustained release of immunotherapeutic agents. The in situ gel vaccine effectively triggers STING-driven tumor vascular normalization and activates DCs and effector CD8^+^ T cells within tumors, as well as in their draining lymph nodes and distal metastases. This results in the suppression of tumor progression and prevention of recurrence in immunologically cold tumors. (B) Nanoscale STING agonist cGAMPnps promotes the efficient uptake of cGAMP and induces activation of the type I IFN response in DCs. The hydrogel formulation containing ICD inducer Dox triggers release of tumor antigens and enhances and prolongs STING activation by cGAMPnps. Furthermore, checkpoint inhibitors (e.g., anti-PD-1 antibody) or immunomodulators (OX40L) incorporated into the hydrogel reprogram the immunosuppressive TME, enhancing the efficacy of in situ cancer vaccination.

In our study, we engineered an in situ gel vaccine using a thermoresponsive silk fibroin (SF) hydrogel encapsulating a nanoscale STING agonist [cGAMP nanoparticle (cGAMPnp)], an ICD inducer, and an immunomodulator. Figure 1 illustrates our design of the in situ gel vaccine in improving the efficacy of STING agonists for bolstered antitumor immunity. Upon administration, the hydrogel transitions from liquid to solid at physiological temperature (37 °C), a change facilitated by the structural properties of SF. This sol–gel transition transforms the structure of SF from random coil to beta-sheet, improving the mechanical stability of the hydrogel and ensuring its retention at the injection site. Consequently, the hydrogel forms a depot, enabling the controlled and prolonged release of the immunotherapeutic agents, aiming for sustained STING activation and enhanced anticancer immunity. Exploiting the advantages of nanocarriers,^30−34^ the cGAMPnps were designed with a core–shell architecture, enveloped by the cationic lipid 1,2-dioleoyl-3-trimethylammonium-propane (DOTAP). This design optimizes cGAMP delivery to DCs,^35^ enhancing their maturation and type I IFN production. Importantly, our study highlights the cooperative effect between ICD-associated damage-associated molecular patterns (DAMPs) and cGAMPnps. This effect amplifies and prolongs STING activation and DC maturation due to the delayed degradation of both cGAMP and the STING. Lastly, the integration of a checkpoint inhibitor (anti-PD-1 antibody) or immunomodulator (OX40L) into the hydrogel reprograms the immunosuppressive tumor microenvironment (TME), further potentiating the efficacy of the in situ cancer vaccination.

The use of this in situ gel vaccine showed promising results in a variety of tumors including triple-negative breast cancer (TNBC), glioblastoma (GBM), hepatocellular carcinoma (HCC), and pancreatic ductal adenocarcinoma (PDAC). Notably, GBM, HCC, and PDAC, often referred to as “immunologically cold” due to their sparse tumor-infiltrating lymphocytes and excessive immunosuppressive cells, have shown potent efficacy with this treatment.^36−39^ This vaccination approach led to STING-induced tumor vascular normalization and a significant increase in DC activation and effector CD8^+^ T cells in both tumors and associated lymph nodes, indicating robust antitumor immunity. Furthermore, this in situ gel vaccine proved to be successful in activating antitumor immunity in the surgical bed and distal metastases, effectively suppressing tumor recurrence and metastasis (Figure 1), marking its potential for clinical cancer treatment.

## Results

### cGAMP-Loaded NPs Facilitate STING Activation and DC Maturation

**Figure 2:**
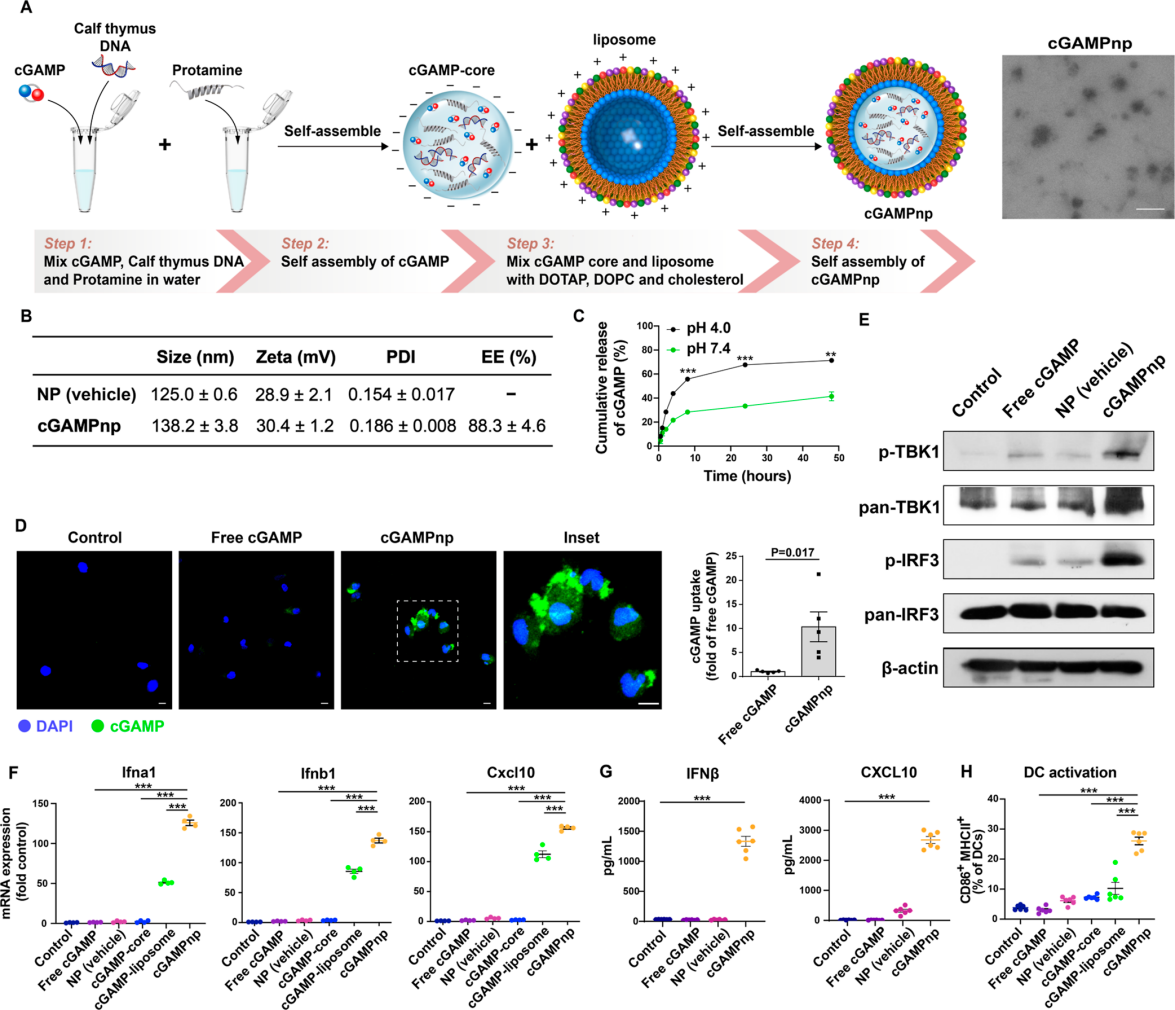
cGAMP-loaded NPs (cGAMPnps) enhance intracellular cGAMP uptake and STING activation in DCs. (A) Schematic illustration of the synthesis procedure and representative TEM image of cGAMPnps. Scale bar, 200 nm. (B) Size, zeta potential, polydispersity index (PDI), and EE of the cGAMPnp or empty NP (vehicle) (*n* = 3). (C) Kinetics of cGAMP release from NPs under physiological (pH 7.4) or acidic (pH 4.0) conditions. The release of cGAMP was measured as the fluorescence intensity of fluorescein-labeled cGAMP (*n* = 3). (D) Fluorescein-cGAMP uptake by bone marrow-derived dendritic cells (BMDCs) treated with cGAMPnps or free cGAMP. The cellular uptake of fluorescein-cGAMP (1 μM) was imaged and quantified using a Zeiss LSM 780 confocal microscope (*n* = 5). (E) Western blotting was utilized to analyze TANK-binding kinase 1 (TBK1) and Interferon Regulatory Factor 3 (IRF3) phosphorylation in BMDCs treated with free cGAMP, empty vehicle, or cGAMPnps (1 μM). (F) mRNA expression levels of type I IFN and inflammation-related genes (*Ifna1*, *Ifnb1,* and *Cxcl10*) in BMDCs 6 h after treatment with free cGAMP, empty vehicle, cGAMP-DNA-protamine core (cGAMP-core), cGAMP loaded in the liposome (cGAMP-liposome), or cGAMPnps (0.5 μM) were measured by RT–qPCR. The results are expressed as the fold change relative to the corresponding level in the untreated control group (*n* = 4). (G) Cytokine secretion from BMDCs was measured by ELISA 24 h after treatment with free cGAMP, empty vehicle or cGAMPnps (0.5 μM) (*n* = 6). (H) Expression of MHC-II and CD86 in CD11c^+^ BMDCs was measured by flow cytometry 24 h after treatment with free cGAMP, empty vehicle, cGAMP-core, cGAMP-liposome, or cGAMPnps (1 μM) (*n* = 6). All data are shown as the mean ± standard error of the mean (SEM) *P < 0.05, **P < 0.01, ***P < 0.001.

To enhance the cellular uptake of cGAMP and STING activation in DCs, we utilized NPs with a core–shell structure to encapsulate cGAMP (Figure 2A). These NPs, designated as cGAMPnps, comprise a core made of DNA and protamine encapsulated within a synthetic lipid bilayer shell. The core is negatively charged calf thymus DNA, employed as a carrier to efficiently package cGAMP in a condensed form, assisted by the presence of positively charged protamine. This core was subsequently enclosed within a positively charged liposome composed of DOTAP (Figure 2A). Transmission electron microscopy (TEM) images show that cGAMPnps were spherical (Figure 2A). cGAMPnp was characterized using dynamic light scattering, which revealed a size of 138.2 ± 3.8 nm, a surface charge of 30.4 ± 1.2 mV, and a polydispersity index (PDI) of 0.186 ± 0.008. The cGAMP encapsulation efficiency (EE) was 88.3 ± 4.6% (Figure 2B). The kinetic profile for the concomitant release of cGAMP from NPs was evaluated under physiological and acidic pH conditions at 37 °C. The release rate of cGAMP from cGAMPnps under acidic conditions (pH 4.0) was significantly higher than that under physiological conditions (pH 7.4) (Figure 2C). This pH-responsive release might be due to the protonation of cGAMP under acidic conditions, which attenuates its interaction with the positively charged protamine, thereby facilitating the release of cGAMP from the NPs. The pH-dependent release of cGAMP signified the controlled and efficient release of cGAMP from NPs in the acidified endosomes/lysosomes of DCs.

We next explored the capacity of cGAMPnps to deliver cGAMP into the DCs. As shown in Figure 2D,E, more fluorescein-cGAMP was taken up by BMDCs when delivered by cGAMPnps as compared to its free form. We subsequently examined its effect on the STING-mediated immune response by accessing activation of the STING signaling pathway and induction of type I IFN signaling. Compared to the free cGAMP or unloaded NPs, cGAMPnps generated a stronger STING-mediated immune response, as indicated by increased TBK1 and IFN Regulatory Factor 3 (IRF3) phosphorylation (Figure 2E) and increased expression of type I IFN and other inflammation-related genes (*Ifna1*, *Ifnb1,* and *Cxcl10*) (Figure 2F). Consistent with mRNA expression, cGAMPnps significantly increased corresponding cytokine secretion from BMDCs (Figure 2G), leading to their enhanced maturation (CD86^+^MHCII^+^), as illustrated in Figure 2H.

In contrast, cGAMP in liposomes without a core–shell structure (cGAMP-liposomes) or in cGAMP-DNA-protamine cores without lipid shells (cGAMP-cores) only mildly increased cGAMP uptake, stimulated type I IFN production, and facilitated DC maturation (Figures 2F–H and S1). Additionally, cGAMPnps demonstrated superior serum stability compared with cGAMP-liposomes or cGAMP-cores (Table S1). Our findings underline that cGAMPnp with dense core–shell structures serves as an effective vehicle for intracellular delivery of cGAMP, activating the STING pathway, promoting type I IFN production in BMDCs, and hence enhancing DC maturation.

### Gene Expression and the Interplay between STING Agonists and ICD-Associated DAMPs in BMDCs

Numerous studies have demonstrated that combining STING agonists with chemotherapy, known as chemo-immunotherapy, significantly enhances the anticancer effects. Yet, the underlying mechanisms by which chemotherapy may potentiate STING-mediated anticancer immunity are not fully understood. To investigate this, we examined a single-cell gene expression data set from primary breast cancer samples treated with Dox, an ICD inducer.^40^ The analysis revealed the upregulation of genes related to IFN signaling (Table S2) and STING-mediated immune responses (Table S3). These findings suggest a possible interaction between Dox-induced ICD and STING-driven anticancer immunity.

**Figure 3:**
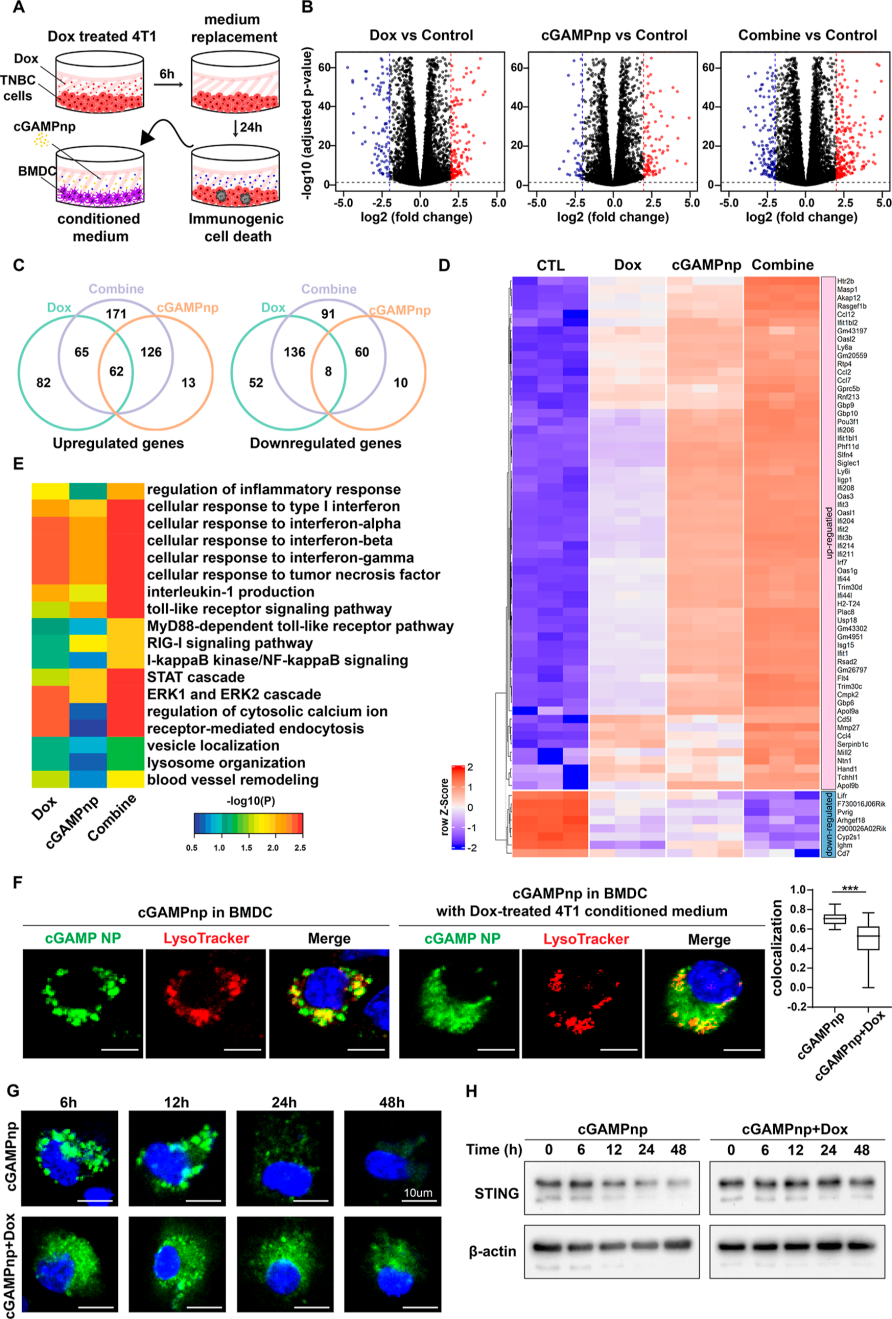
Gene expression changes and ontological analysis in BMDCs treated with cGAMPnps and ICD-associated DAMPs. (A) Schematic representation of the experimental design. 4T1 cells were treated with Dox (2 μM) for 6 h, the medium was replaced with fresh medium, and the conditioned medium was collected after 24 h. BMDCs were treated with cGAMPnps (1 μM) alone or with the collected the conditioned medium. (B) Heatmap showing the differentially expressed genes in BMDCs upon treatment with cGAMPnps, ICD-associated DAMPs from Dox-treated 4T1 cells (Dox), or the combination of both compared to the untreated control group (*n* = 3). (C) Venn diagram showing the number of differentially expressed genes among different treatment groups. (D) Heatmap showing the expression profiles of common genes among different treatment groups (*n* = 3). (E) Gene ontological analysis of differentially expressed genes related to inflammatory and STING pathway activation (*n* = 3). (F) Confocal microscopy images of BMDCs treated with cGAMPnps (green) alone or in combination with ICD-associated DAMPs and stained with Lysotracker Red DND-99 (red). BMDCs were treated with or without the conditioned medium from 4T1 breast cancer cells exposed to Dox for 24 h and then incubated with cGAMPnps loaded with fluorescein-cGAMP for 6 h; subsequently, the uptake and trafficking of cGAMP in BMDCs was examined. Scale bar, 10 μm. The colocalization of fluorescein-cGAMP with Lysotracker Red DND-99 in BMDCs was quantified (*n* = 15). Data are shown as the mean ± SEM ***P < 0.001. (G) BMDCs treated by cGAMPnps with or without the conditioned medium from 4T1 breast cancer cells exposed to Dox exhibit different temporal patterns of cGAMP-punctate formation and depletion. Scale bar, 10 μm. (H) Western blot analysis of STING degradation in BMDCs treated with cGAMPnp (1 μM) alone or in combination with ICD-associated DAMPs from Dox-treated 4T1 cells (Dox) for different time points. All data are shown as the mean ± SEM.

To understand how ICD-associated DAMPs affect cGAMP-mediated STING activation, we investigated changes in gene expression in BMDC cells treated under various conditions. Specifically, transcriptome analysis of BMDCs treated with cGAMPnps with or without conditioned medium from cultured 4T1 breast cancer cells exposed to the ICD inducer Dox (*n* = 3/group) was performed (Figure 3A). In comparison with no treatment, exposure to ICD-associated DAMPs released from Dox-treated 4T1 cells and cGAMPnps alone caused significant changes in gene expression: the expression of 405 (196 downregulated, 209 upregulated) and 279 (78 downregulated, 201 upregulated) genes, respectively, was altered (Figure 3B, Appendix). These effects were more profound in the BMDCs treated with cGAMPnps and ICD-associated DAMPs from Dox-treated 4T1 cells, where 719 (295 downregulated, 424 upregulated) genes were differentially expressed compared to their expression in the untreated control group (Figure 3B). We then used Venn diagrams to visualize the numbers of differentially expressed genes shared among and specific to the different treatment groups. Our analyses indicated that combination treatment with cGAMPnps and conditioned medium from Dox-treated 4T1 cells significantly changed the expression profiles of 262 genes (171 up-regulated and 91 down-regulated) whose expression was not significantly affected by exposure to either ICD-associated DAMPs or cGAMPnp alone (Figure 3C). In addition, there were 70 (62 upregulated and 8 downregulated) common differentially expressed genes among the single and combination treatment groups. Most of these common differentially expressed genes also exhibited a more pronounced change in expression upon combination treatment (Figure 3D). Our study suggests the potential of combined treatments in significantly altering the molecular landscape of BMDCs.

We next performed gene ontology analysis to evaluate the changes in gene clusters related to inflammation and STING activation in BMDCs subjected to different treatments. We observed that the treatment with cGAMPnps combined with ICD-associated DAMPs elevated not only type I IFN and NF-κB pathways but also other inflammatory pathways, such as Toll-like receptor, RIG-I, STAT, and intracellular calcium regulation pathways. These pathways collectively contributed to a more significant activation of STING compared to the activation observed with the cGAMPnp alone (Figure 3E and Table S4). Moreover, endocytosis, vesicle localization, and lysosome organization pathways involved in cGAMP and STING trafficking or degradation, ERK1/ERK2-mediated DC survival, and blood vessel remodeling involved in reprograming tumor vasculature were upregulated upon combination treatment (Figure 3E). These results suggest that ICD-associated DAMPs could be potential targets for the development of STING-mediated immunotherapy.

Given the potential impact of altered endocytosis pathways and vesicle distribution on STING trafficking and degradation, we then investigated the intracellular dynamics of fluorescein-labeled cGAMP in BMDCs upon treatment with cGAMPnps in response to ICD-associated DAMPs (Figure 3F,G).^41^ We observed that when delivered by cGAMPnps, cGAMP formed distinct puncta within cells as early as 6 h post-treatment, followed by rapid degradation at 24 h (Figure 3F,G). By contrast, exposure to DAMP-enriched conditioned medium from Dox-treated 4T1 cells delayed degradation of cGAMP, as indicated by the reduced fusion of cGAMP-puncta with lysosomes (Figure 3F,G). This reduced fusion time led to a subsequent delay in the degradation of cGAMP and STING within the BMDCs (Figure 3G,H). Consistently, endogenous STING was rapidly degraded following the treatment with cGAMPnps (Figure 3H). However, the presence of a DAMP-containing conditioned medium delayed STING degradation in BMDCs (Figure 3H).

### ICD-Associated DAMPs Cooperate with cGAMP to Boost and Extend STING Activation in BMDCs

**Figure 4:**
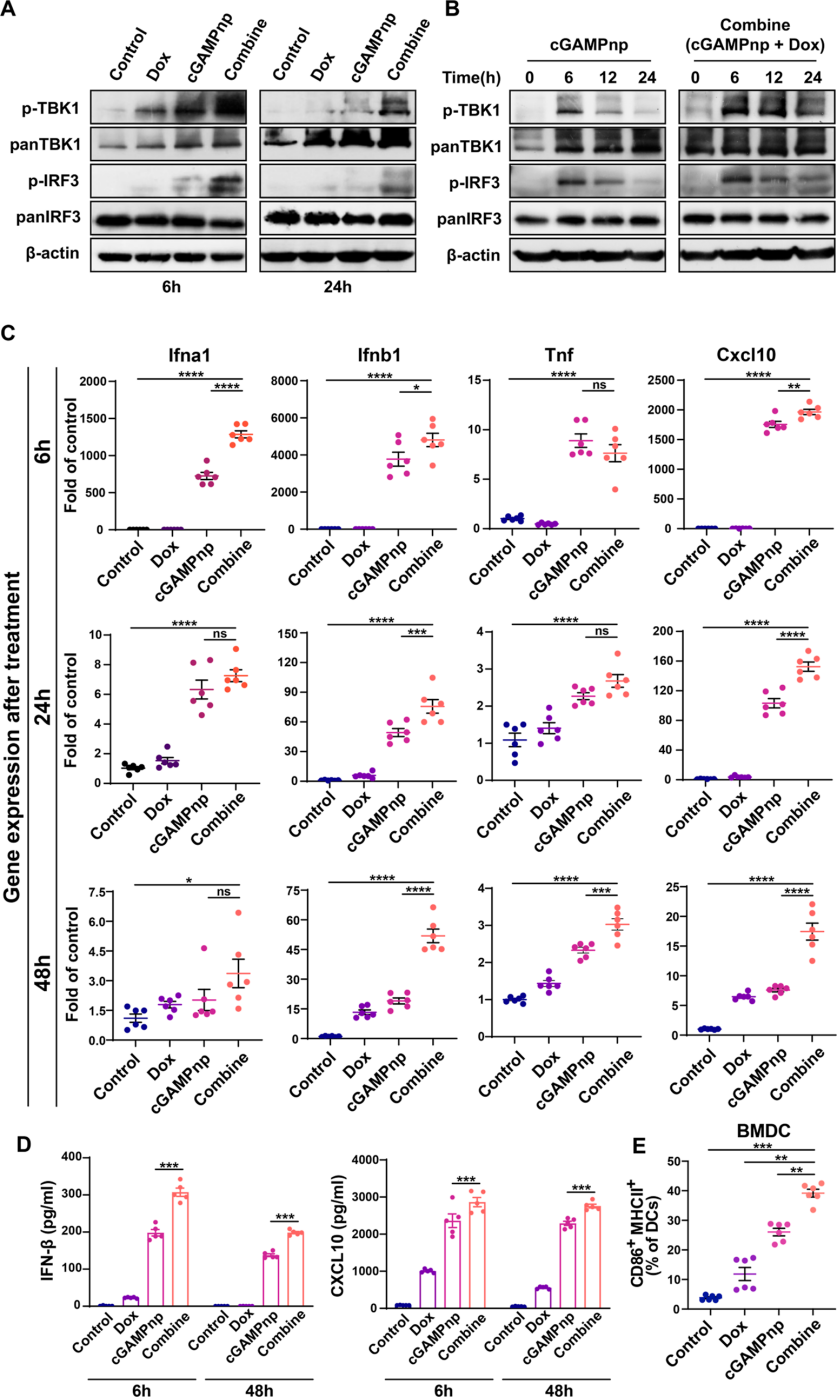
Enhanced and prolonged STING activation in BMDCs treated with cGAMPnps and ICD-associated DAMPs. (A) Western blot analysis of TBK1 and IRF3 phosphorylation in BMDCs treated with cGAMPnp (1 μM), ICD-associated DAMPs from Dox-treated 4T1 cells (Dox), or the combination of both for 6 or 24 h, as described in Figure 3a. (B) Western blot analysis of TBK1 and IRF3 phosphorylation in BMDCs treated with cGAMPnp (1 μM) alone or in combination with ICD-associated DAMPs from Dox-treated 4T1 cells (Dox) for different time points. (C) mRNA expressions of type I IFN and inflammation-related genes (*Ifna1*, *Ifnb1*, *Tnf,* and *Cxcl10*) in BMDCs 6, 24, or 48 h after treatment with cGAMPnp (1 μM), ICD-associated DAMPs from Dox-treated 4T1 cells (Dox), or the combination of both were measured by RT–qPCR. The results are expressed as the fold change relative to the corresponding level in the untreated control group (*n* = 6). (D) ELISA analysis of IFN-β and CXCL10 production in BMDCs treated with cGAMPnp (1 μM), ICD-associated DAMPs from Dox-treated 4T1 cells (Dox), or the combination of both for 6 or 24 h (*n* = 5). (E) Flow cytometry analysis of MHC II and CD86 expression in BMDC cells treated with cGAMPnp (1 μM), ICD-associated DAMPs from Dox-treated 4T1 cells (Dox), or the combination of both for 24 h (*n* = 6). ns, not significant. All data are shown as the mean ± SEM *P < 0.05, **P < 0.01, ***P < 0.001, ****P < 0.0001.

To further understand if these mechanisms could generate sustained STING activation, we next examined the impact of ICD-associated DAMPs from Dox-treated 4T1 cells on the kinetics and magnitude of the STING-mediated immune response. cGAMPnps triggered a potent STING-mediated immune response, evidenced by increased TBK1 and IRF3 phosphorylation (Figure 4A,B) and upregulation of inflammation-related genes (Figure 4C) around 6 h post-treatment, swiftly followed by a decline in immunity (Figure 4A–C). BMDCs in DAMP-containing medium from Dox-treated 4T1 cells extended cGAMP-mediated STING activation, as evidenced by greater and prolonged TBK1 and IRF3 activation, increased expression of type I IFN-related genes, and production of inflammatory cytokines over extended periods (6–48 h) (Figure 4A–D), leading to enhanced DC maturation (CD86^+^MHCII^+^) (Figure 4E). The effect was similar when BMDCs were exposed to other ICD inducers, such as oxaliplatin (Figure S2).

Additionally, we investigated whether DAMP molecules cooperate with cGAMP to enhance and prolong the STING activation. Notably, HMGB1 plays a key role in preventing STING degradation. We observed that the stability of cGAMP in BMDCs and the expression of type I IFN-related molecules, enhanced by ICD-associated DAMPs from Dox-treated 4T1 cells, were reduced when HMGB1 was downregulated using siRNA (Figure S3). This suggests that HMGB1, a major DAMP induced by Dox, is crucial for maintaining STING activation. Overall, these findings highlight that combining cGAMPnps with ICD inducers can extend and intensify immune responses.

### Sustained Release of Dox and cGAMPnps from SF Hydrogels Enhances STING Activation in TNBC

**Figure 5:**
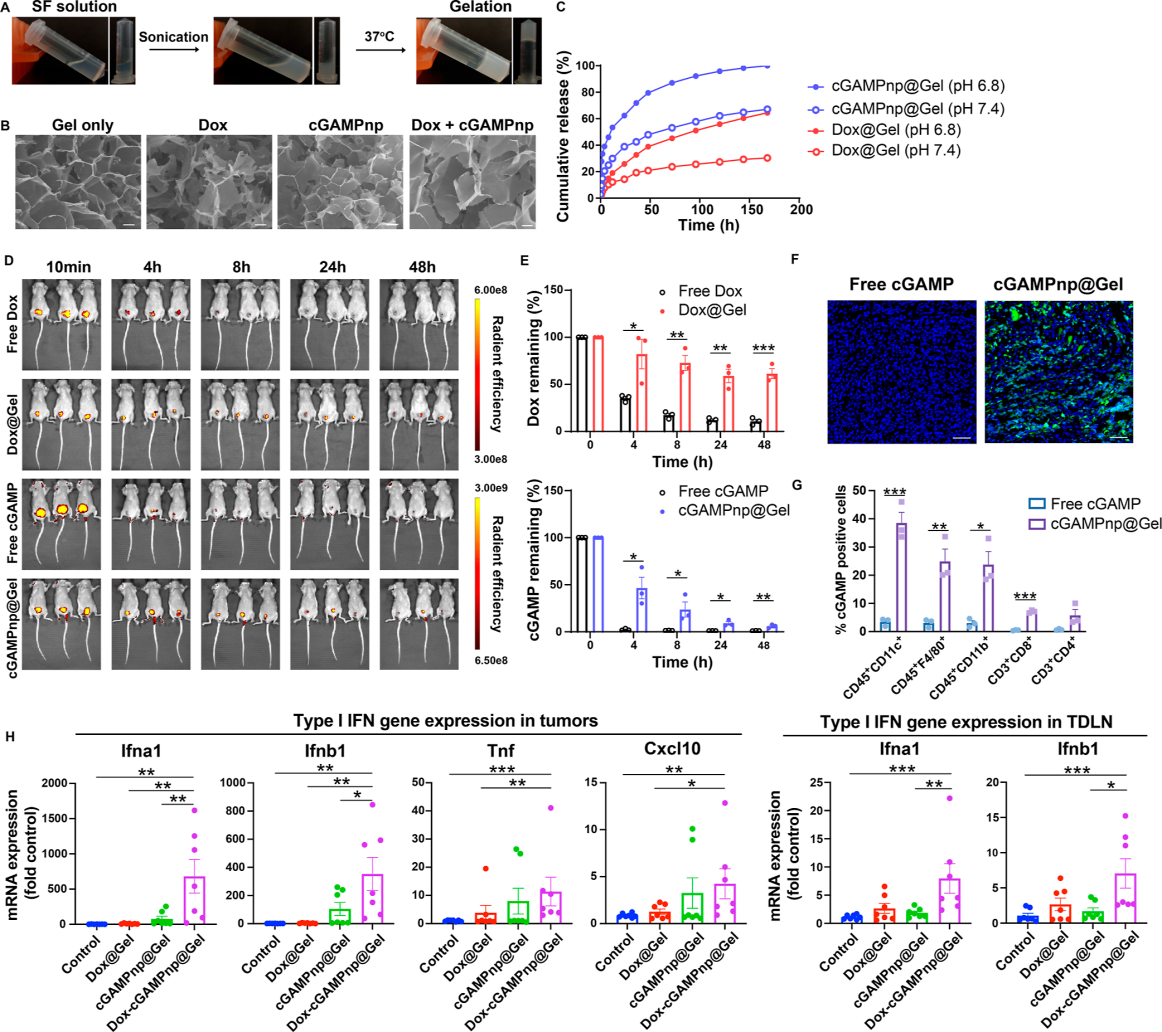
Injectable SF hydrogels enable a sustained release of cGAMPnps and Dox to potentiate STING activation in TNBC tumors. (A) Photographs of SF in various stages of the gelation process. (B) SEM images of the SF hydrogel before and after loading with cGAMPnps and Dox. (C) Release of fluorescein-cGAMP loaded cGAMPnp and Dox from SF hydrogels under physiological conditions (pH 7.4) and acidic conditions (pH 6.8) (*n* = 3). (D) In vivo fluorescent imaging of 4T1 tumor-bearing mice at 4, 8, 24, and 48 h after intratumoral injection of free Dox, free fluorescein-cGAMP or Dox, or fluorescein-cGAMPnp loaded SF hydrogels. (E) Quantification analysis of the retention of Dox and fluorescein-cGAMP in tumor tissues after injection of free drugs or hydrogels (*n* = 3). (F) Representative confocal images of the intracellular uptake of cGAMP by tumors. Green, cGAMP; blue, DAPI. Scale bar, 50 μm. (G) Flow cytometry was used to analyze the immune cell types that take up fluorescein-cGAMP in the 4T1 tumors 2 h after injection of free fluorescein-cGAMP or fluorescein-cGAMPnp-loaded SF hydrogels. DCs, macrophages, and monocytes were identified as CD45^+^CD11c^+^, CD45^+^F4/80^+^, and CD45^+^CD11b^+^ cells, respectively, and other lymphocytes were identified as CD3^+^CD4^+^ cells and CD3^+^CD8^+^ cells. (H) mRNA expressions of type I IFN and inflammation-related genes (*Ifna1*, *Ifnb1*, *Tnf*, and *Cxcl10*) in 4T1 tumors and TDLNs 24 h after intratumoral injection of Dox@Gel, cGAMPnp@Gel, or Dox-cGAMPnp@Gel (*n* = 7). All data are shown as the mean ± SEM *P < 0.05, **P < 0.01, ***P < 0.001.

To achieve a controlled and sustained release of immune-stimulating agents in tumor sites for in situ cancer vaccination, SF hydrogels with excellent biocompatibility and controllable degradation were utilized to load cGAMPnps and the ICD inducer Dox.^42−44^ cGAMPnps and Dox were incorporated into injectable thermoresponsive SF hydrogels, allowing for their implantation via minimally invasive injection. These hydrogels were further incubated at 37 °C for 5 min to mimic physiological conditions to facilitate the sol–gel transition and improve the mechanical properties of the gel (Figure 5A). SEM revealed that the porosity and nature of the hydrogel structure remained similar after the encapsulation of cGAMPnps and Dox (Figure 5B). The Dox- and cGAMPnp-loading efficiencies of the SF hydrogels were 99.67% and 94.99%, respectively. The cumulative release of cGAMPnps was then assessed using fluorescein-cGAMP. Continuous release of Dox and cGAMPnps from the SF hydrogels occurred over a period of 7 days (Figure 5C), suggesting that the hydrogels achieve sustained release of both nanoscale and small-molecule immunostimulatory agents. Furthermore, SF hydrogels showed a faster release of therapeutic cargoes (Dox and cGAMPnps) in acidic conditions (pH 6.8), similar to the TME’s acidity, compared to neutral conditions (pH 7.4). This is due to weaker hydrogen bonds between SF and cargoes at lower pH, enabling an efficient localized release in the tumor bed.^45^

Notably, we demonstrated that Dox-loaded SF hydrogels induced a higher toxicity in 4T1 breast cancer cells than in BMDCs, highlighting their potential for targeted cancer therapy. (Figure S4).

To assess the potential of the SF hydrogels to act as a drug delivery scaffold, we compared tumoral injections of Dox or fluorescein-labeled cGAMPnp solutions and Dox or fluorescein-labeled cGAMPnp-loaded SF hydrogels (Dox@Gel or cGAMPnp@Gel) in the 4T1 orthotopic TNBC model, a model chosen for its high malignancy and low immunogenicity, thus reflecting challenging clinical scenarios. The subsequent release profiles from tumor tissues revealed notable differences (Figure 5D,E). Mice treated with free drugs lost over 80% of fluorescence within 8 h postinjection, whereas hydrogel-treated mice maintained a strong fluorescence signal, with approximately 60% of Dox and 10% of cGAMP present in the tumor tissue 2 days after injection (Figure 5E). Moreover, cGAMP delivered via cGAMPnp@Gel demonstrated a superior intracellular uptake in the tumor tissue compared to free cGAMP (Figure 5F), primarily taken up by CD45^+^CD11c^+^ DCs, CD45^+^F4/80^+^ macrophages, and CD45^+^CD11b^+^ monocytes (Figure 5G). These results suggest that the SF hydrogels prolong the therapeutic agent retention and enhance their uptake within the tumor bed.

To test whether Dox enhanced or prolonged the STING activation induced by cGAMPnps in vivo, we intratumorally injected hydrogels loaded with cGAMPnps (20 μg/mouse) and Dox (50 μg/mouse) (Dox-cGAMPnp@Gel) into 4T1 tumors and measured the expression of type I IFN-related genes in the tumors 24 h after injection. Consistent with the in vitro results, Dox and cGAMPnps (Dox-cGAMPnp@Gel) synergistically increased STING activation in tumors and tumor draining lymph nodes (TDLN), as indicated by the increased expression of type I IFN-related genes (*Ifna1*, *Ifnb1*, *Tnf*, and *Cxcl10*) compared with their expression upon treatment with cGAMPnps (cGAMPnp@Gel) or Dox (Dox@Gel) alone (Figure 5H).

### In Situ Gel Vaccine Induces Potent Antitumor Immunity in Orthotopic TNBC Models

**Figure 6:**
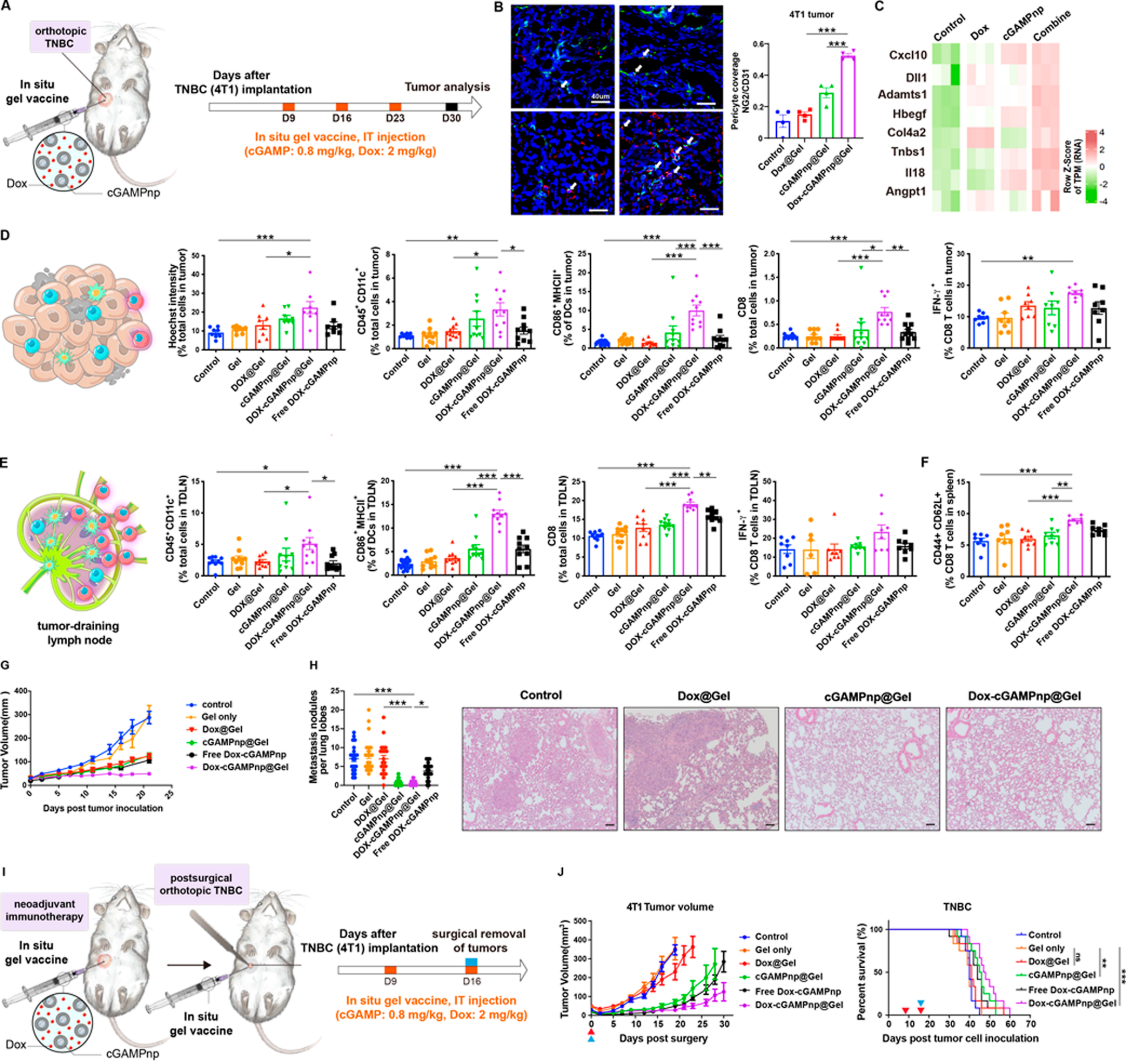
Effect of the in situ gel vaccine (Dox-cGAMPnp@Gel) on vessel normalization and activation of anticancer immunity in an orthotopic 4T1 TNBC model. (A) Schematic of the experimental protocol. Various formulations were intratumoral administered on days 9, 16, and 23 after the orthotopic inoculation of 4T1 cells, and tumors and TDLNs were analyzed on day 30. (B) Pericyte coverage in TNBC was imaged and quantified using a Zeiss LSM 780 confocal microscope (*n* = 4). CD31-positive endothelial cells are stained green, and NG2-positive pericytes are stained red. Scale bar, 40 μm. (C) Expression of the antiangiogenic and vessel stabilizing factors in BMDCs after treatment with cGAMPnps with the conditioned medium from Dox-treated 4T1 cells measured through RNA-seq (*n* = 3). (D) Hoechst 33342-positive cells (*n* = 8), DCs (CD45^+^CD11c^+^), activated DCs (CD86^+^MHCII^+^), and cytotoxic CD8^+^ T lymphocytes (CD3^+^CD8^+^) in tumors were detected by flow cytometry (*n* = 10). (E) DCs (CD45+CD11c+), activated DCs (CD86^+^MHCII^+^), and cytotoxic CD8^+^ T lymphocytes (CD3^+^CD8^+^) in TDLNs were detected by flow cytometry (*n* = 10). (F) Percentages of central memory (CD44^+^CD62L^+^) CD8 T cells in spleens were detected by flow cytometry (*n* = 8). (G) Tumor growth curve of the 4T1 TNBC model after treatment with various formulations (*n* = 8). (H) Number of spontaneously occurring lung metastatic nodules in the orthotopic TNBC (4T1) model was reduced in mice treated with cGAMPnp@Gel or Dox-cGAMPnp@Gel (*n* = 21). Images of hematoxylin and eosin (H&E) staining show metastatic tumor nodules in the lung. Scale bar, 200 μm. (I) Schematic of the experimental protocol. Nine days after the orthotopic inoculation of 4T1 cells, mice were intratumorally administered with various formulations. The implanted tumor was surgically removed and various formulations were injected into the surgical site on day 16. (J) Tumor growth curve and overall survival in the postsurgical 4T1 TNBC model after treatment with various formulations (*n* = 12). The blue arrow shows surgical removal of primary tumors and the red arrow shows administration of various formulations. A comparison of survival curves was performed using a log-rank Mantel–Cox test (two-sided). All data are shown as the mean ± SEM *P < 0.05, **P < 0.01, ***P < 0.001.

We then evaluated whether the enhanced STING activation induced by Dox-cGAMPnp@Gel modulated the TME and augmented the immune response in the 4T1 orthotopic TNBC model after the intratumoral injection of different formulations (Figure 6A). The transcriptome analysis of BMDCs revealed that treatment with cGAMPnps and DAMPs from Dox-treated 4T1 cells led to significant alterations in genes related to blood vessel remodeling, as shown in Figure 3E. This finding prompted us to explore how STING activation by Dox-cGAMPnp@Gel might influence tumor vascular remodeling. The hydrogel loaded with both Dox and cGAMPnps (Dox-cGAMPnp@Gel) normalized tumor vasculatures in 4T1 tumors indicated by increased pericyte coverage of tumor vessels (NG2^+^ CD31^+^), compared with cGAMPnps or Dox alone in the hydrogel (Figure 6B). These may be mediated by upregulation of type I IFN genes (Figures 4C and 5H) and vascular stabilizing genes (e.g., Cxcl10, DLL1, Hbegf, Angpt1, and Col4a; Figure 6C). Consequently, the hydrogel loaded with both Dox and cGAMPnps (Dox-cGAMPnp@Gel) substantially increased tumor perfusion (Hoechst 33342^+^) (Figure 6D), the proportions and activation of DCs and cytotoxic T cells within tumors and TDLNs (Figure 6D,E), and the proportions of CD62L^+^CD44^+^ central memory CD8 T cells in spleens (Figure 6F), when compared to no treatment control, the empty Gel, cGAMPnp@Gel, Dox@Gel, or free drugs, leading to the complete suppression of tumor growth and distal lung metastasis in the orthotopic 4T1 breast tumor model (Figure 6G,H). In contrast, the empty Gel had no effect on tumor growth, and DOX@Gel, cGAMPnp@Gel, and free drugs only moderately inhibited tumor growth in the orthotopic 4T1 breast tumor model (Figure 6D–H). In summary, the in situ gel vaccine (Dox-cGAMPnp@Gel) modulates TME and amplifies antitumor immune responses, effectively suppressing tumor growth and metastasis in the 4T1 orthotopic TNBC model.

Moreover, recent studies have demonstrated that neoadjuvant immunotherapy in combination with surgery can enhance the long-term prognosis of TNBC. In light of this, we further assessed the efficacy of the in situ gel vaccine, Dox-cGAMPnp@Gel, within a neoadjuvant immunotherapy setting combined with surgery, aimed at preventing cancer recurrence post-tumor resection in the 4T1 orthotopic TNBC model. Seven days after administering Dox-cGAMPnp@Gel, we surgically excised the 4T1 tumors and then reapplied the hydrogel near the removal site (Figure 6I). The Dox-cGAMPnp@Gel notably slowed tumor growth in the clinically relevant postsurgical TNBC model compared to the empty Gel, DOX@Gel, or cGAMPnp@Gel (Figure 6J). While it improved overall survival, relapse-free survival was not achieved with Dox-cGAMPnp@Gel (Figure 6J). Considering the observed moderate activation of cytotoxic T cells in the tumors and TDLNs post-treatment (Figure 6D,E), it appears that the immunosuppressive TME might be attenuating the anticancer immune response induced by the in situ gel vaccine.

Given that PD-L1, extensively expressed in many tumors and inherently in 4T1 tumors (Figure S5), inhibits T-cell activity, the application of anti-PD-1 antibodies to restore T-cell function is valuable.^46,47^ Moreover, OX40L enhances T-cell expansion and survival, boosting their anticancer response.^48^ Notably, we observed an increase in PD-1 and OX40 positive CD8 T cells in TDLNs and primary metastatic organ lungs with orthotopic implantation of 4T1 cells, which overexpressed PD-L1 (Figure S5). To overcome the immunosuppressive TME and potentiate the STING-driven antitumor response, we propose integrating the PD-1 blockade or the OX40L activation with Dox-cGAMPnp@Gel.

### In Situ Gel Vaccine Induces Potent Antitumor Immunity in Postsurgical TNBC and GBM Models

**Figure 7:**
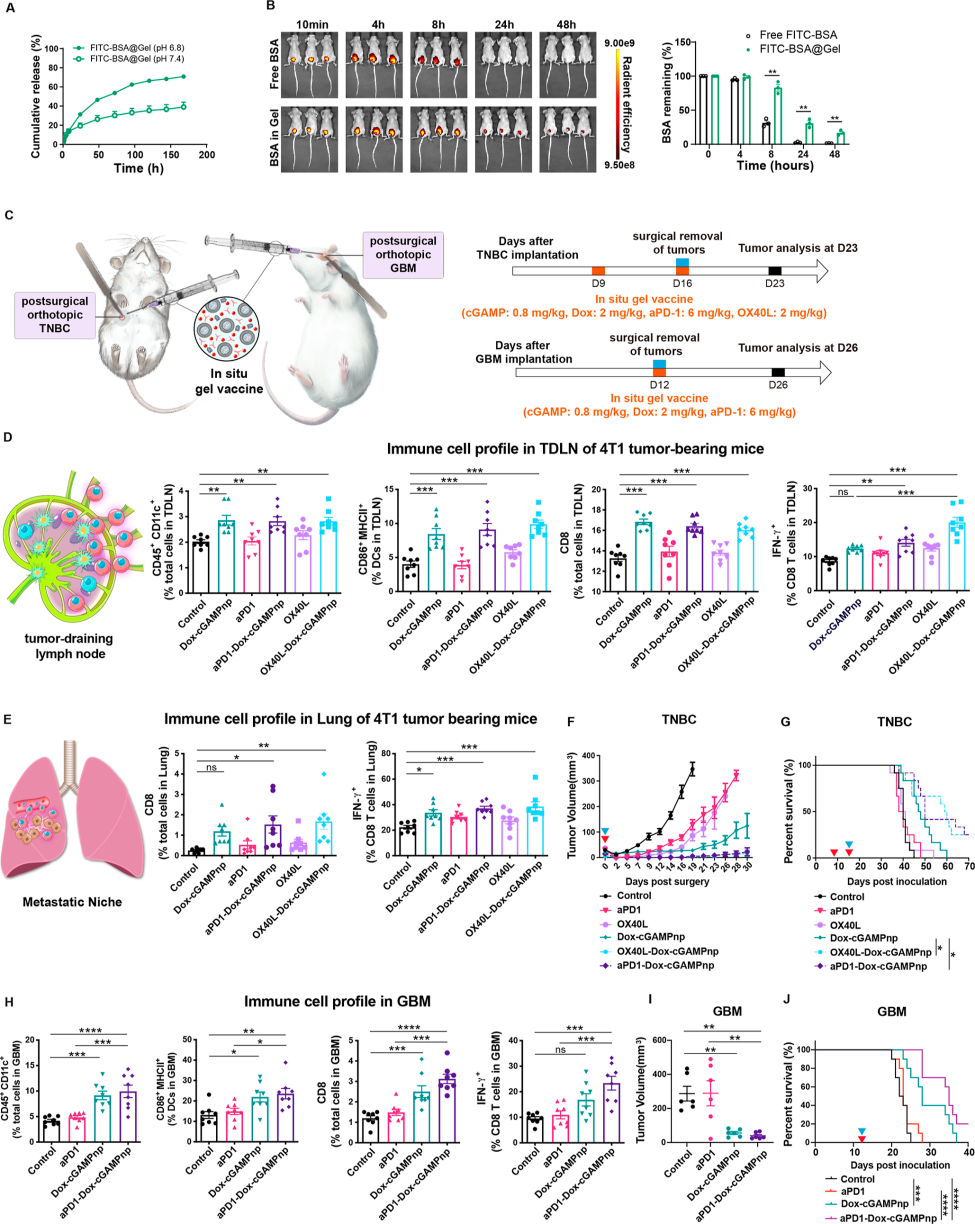
In situ gel vaccine containing cGAMPnps, Dox, and an immunomodulator (anti-PD-1 antibody or OX40L) achieves potent antitumor immunity and prevents postsurgical tumor recurrence in orthotopic TNBC and GBM models. (A) Release of FITC-BSA from SF hydrogels under physiological conditions (pH 7.4) and acidic conditions (pH 6.8) (*n* = 3). (B) In vivo fluorescent imaging of 4T1 tumor-bearing mice at 4, 8, 24, and 48 h after intratumoral injection of free FITC-BSA, or FITC-BSA-loaded SF hydrogels. Quantification analysis of the retention of FITC-BSA in tumor tissues (*n* = 3). (C) Schematic of the experimental protocol. For the orthotopic TNBC model, 9 days after the orthotopic inoculation of 4T1 cells, mice were intratumorally administered with various formulations. The implanted tumor was surgically removed, and various formulations were injected into the surgical site on day 16, and tumors and TDLNs were analyzed on day 23. For the orthotopic GBM model, 12 days after the orthotopic inoculation of ALTS1C1 cells, the implanted tumor was surgically removed and various formulations were injected into the surgical site. Tumors were analyzed on day 26. (D) DCs (CD45^+^CD11c^+^), activated DCs (CD86^+^MHCII^+^), cytotoxic CD8^+^ T lymphocytes (CD3^+^CD8^+^), and activated CD8^+^ T lymphocytes (IFN-γ^+^ in CD8^+^) in TDLNs of 4T1 tumor-bearing mice were detected by flow cytometry (*n* = 8). (E) Cytotoxic CD8^+^ T lymphocytes (CD3^+^CD8^+^) and activated CD8^+^ T lymphocytes (IFN-γ^+^ in CD8^+^) in lungs of 4T1 tumor bearing mice were detected by flow cytometry (*n* = 8). (F) Growth curve shows the primary tumor regrowth postresection in the 4T1 TNBC model. (G) Overall survival in the 4T1 TNBC model (*n* = 12). (H) DCs (CD45^+^CD11c^+^), activated DCs (CD86^+^MHCII^+^), cytotoxic CD8^+^ T lymphocytes (CD3^+^CD8^+^), and activated CD8^+^ T lymphocytes (IFN-γ^+^ in CD8^+^) in ALTS1C1 GBM were detected by flow cytometry (*n* = 5). (I) Tumor volume in the GBM model in different treatment groups (*n* = 7). (J) Overall survival in the GBM model (*n* = 10). The blue arrow shows surgical removal of primary tumors, and the red arrow shows administration of various formulations. A comparison of survival curves was performed using a log-rank Mantel–Cox test (two-sided). All data are shown as the mean ± SEM *P < 0.05, **P < 0.01, ***P < 0.001.

To enhance the immune stimulation with the in situ gel vaccine, an immune checkpoint inhibitor, anti-PD-1 antibody, or an immune modulator, OX40L, was effectively integrated into the SF hydrogels. The loading efficiencies of anti-PD-1 antibody and OX40L into the SF hydrogels were 96.03% and 95.92%, respectively. Using FITC-labeled BSA as a tracer protein cargo, similar to the results for Dox and cGAMPnps, we observed continuous release of protein cargoes from the SF hydrogels over a period of 5 days (Figure 7A), and the release of protein cargoes under physiological conditions (pH 7.4) was slower than that under acidic conditions (pH 6.8) (Figure 7A). We then compared tumoral injection of free FITC-labeled BSA and FITC-labeled BSA-loaded SF hydrogels in the orthotopic 4T1 breast tumor model. Similar to the results for Dox and cGAMPnps, in free BSA-treated mice, more than 70% of fluorescence was lost within 8 h after injection (Figure 7B), while in hydrogel-treated mice, a strong fluorescence signal could still be detected 2 days after injection, with approximately 15% of FITC-labeled BSA remaining in the tumor tissue (Figure 7B). Our results indicate that the SF hydrogel achieves pH-responsive release of macromolecule protein-based immune-modulating agents and prolongs the retention of these agents like anti-PD-1 antibodies or OX40L within the tumor bed.

We further investigated whether co-encapsulation of the therapeutic anti-PD-1 antibody or OX40L in the hydrogel could enhance the effects of Dox and cGAMPnp in a neoadjuvant immunotherapy setting paired with surgery, aiming to generate a potent immune response and prevent cancer recurrence post tumor resection (Figure 7C). Our findings demonstrate that Dox-cGAMPnp@Gel with or without anti-PD-1 antibody or OX40L in the gel significantly increased the proportion (CD45^+^CD11c^+^) and activation (CD86^+^MHCII^+^) of DCs in the TDLNs compared to surgery alone (Figure 7D). While Dox-cGAMPnp@Gel alone boosted the population of cytotoxic T cells in the TDLNs, the activation of T cells (CD8^+^IFN-γ^+^) remained moderate (Figure 7D). However, when we incorporated the anti-PD-1 antibody or OX40L into the in situ gel vaccine (aPD1-Dox-cGAMPnp@Gel or OX40L-Dox-cGAMPnp@Gel), it significantly improved both the proportion and activation of CD3^+^CD8^+^ cytotoxic T cells in the TDLNs, compared with surgery alone (Figure 7D). Our result highlights the potential of combining anti-PD-1 antibody or OX40L in the in situ gel vaccine to enhance the antitumor immunity.

To assess the feasibility of utilizing this in situ vaccine for metastasis treatment, we next evaluated whether the in situ vaccine-activated T cells could efficiently home to the distal premetastatic lung. Our results show a moderate increase in both the proportion and activation of CD3^+^CD8^+^ cytotoxic T cells in the lungs of mice treated with Dox-cGAMPnp@Gel (Figure 7E). In contrast, the addition of anti-PD-1 antibody or OX40L to the in situ gel vaccine significantly increased both the proportion and the activation of CD3^+^CD8^+^ cytotoxic T cells in the premetastatic lung compared with those upon surgery alone (Figure 7E). Neither the anti-PD-1 antibody nor the OX40L, when individually integrated into the hydrogel (aPD1@Gel or the OX40L@Gel), exhibited substantial effects on the DCs or T cells within the TDLNs and lungs (Figure 7D,E). As a result, when either aPD1-Dox-cGAMPnp@Gel or OX40L-Dox-cGAMPnp@Gel was administered, tumor growth was significantly slower than that instigated by administering hydrogel formulations comprising the anti-PD-1 antibody alone, OX40L alone, or Dox-cGAMPnp@Gel (Figure 7F). With treatment of either aPD1-Dox-cGAMPnp@Gel or OX40L-Dox-cGAMPnp@Gel, 25% demonstrated complete tumor control and achieved disease-free survival within 70 days (Figure 7G). Collectively, our findings suggest that the in situ gel vaccine, enriched with Dox, cGAMPnps, and either anti-PD-1 antibody or OX40L, can stimulate robust antitumor immunity, inhibit metastasis, and prevent cancer recurrence in the postsurgical 4T1 TNBC model.

Given the observed effects of our in situ gel vaccine on inhibiting tumor recurrence following resection, we proceeded to investigate its efficacy in GBM, a disease known for its significant challenges in preventing recurrence after surgical intervention (Figure 7C). To establish an orthotopic GBM surgical model, ALTS1C1 cells were intracranially implanted in mice, and tumors were surgically removed on day 12 postinoculation. Administration of Dox-cGAMPnp@Gel, with or without coloaded anti-PD-1 antibody, near the excision site significantly increased proportions of DCs and cytotoxic T cells in tumors (Figure 7H). The activation of DCs in the treated tumors was significantly enhanced compared to that of surgery alone (Figure 7H). More importantly, the addition of anti-PD-1 antibody to the in situ gel vaccine (aPD1-Dox-cGAMPnp@Gel) profoundly activated cytotoxic T cells in tumors (Figure 7H). Consequently, aPD1-Dox-cGAMPnp@Gel induced a potent antitumor immune response, suppressing tumor growth (Figure 7I) and extending overall survival (Figure 7J) in the postsurgical GBM model. Our findings highlight the potential of the in situ gel vaccine as a promising strategy for both neoadjuvant and adjuvant immunotherapies, when paired with surgical intervention.

### In Situ Gel Vaccine Suppresses Tumor Progression in Orthotopic HCC and PDAC Murine Models and Activates the Immune Response in Human Tissues

**Figure 8:**
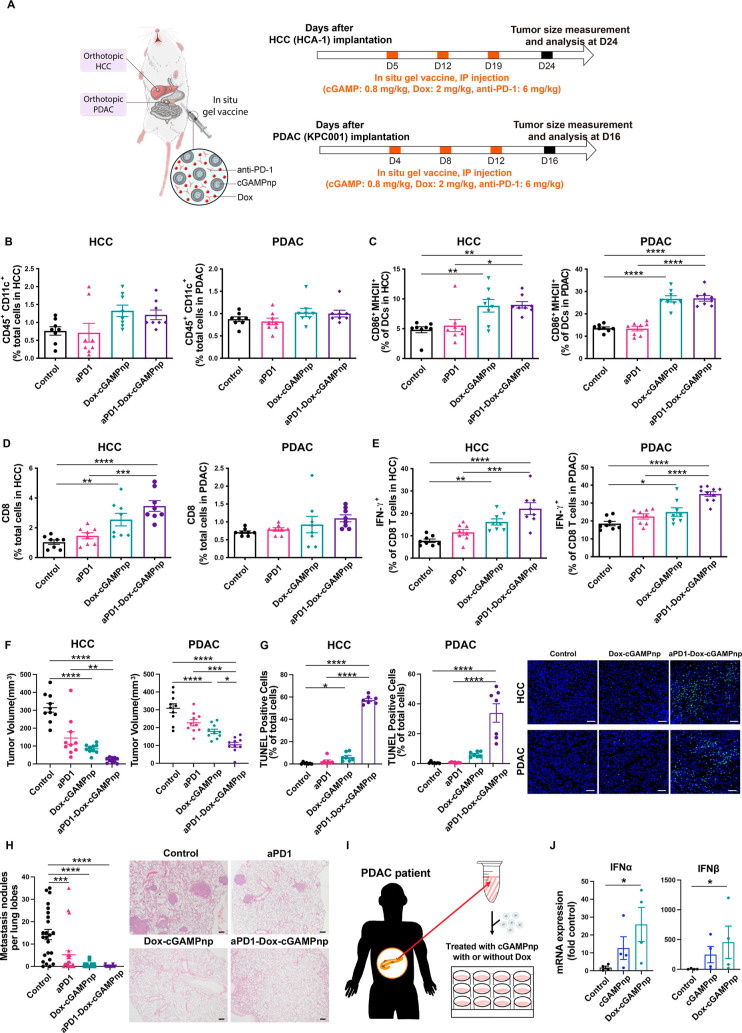
In situ gel vaccine containing cGAMPnp, Dox, and anti-PD-1 antibody achieves potent antitumor immunity in orthotopic HCC and PDAC models. (A) Schematic of the experimental protocol. Five days after the orthotopic implantation of murine HCC (HCA-1) cells, mice were treated with various formulations on days 5, 12, and 19, and tumor size was measured on day 24. Four days after the orthotopic implantation of murine PDAC (KPC001) cells, mice were treated with various formulations on days 4, 8, and 12, and tumor size was measured on day 16. cGAMP (0.8 mg/kg), Dox (2 mg/kg), and anti-PD-1 antibody (6 mg/kg) loaded in SF gels were intraperitoneally injected into orthotopic HCC (HCA-1) and PDAC (KPC001) models. (B–E) DCs (CD45^+^CD11c^+^) (B), activated DCs (CD86^+^MHCII^+^) (C), cytotoxic CD8^+^ T lymphocytes (CD3^+^CD8^+^) (D), and activated CD8^+^ T lymphocytes (IFN-γ^+^ in CD8^+^) (E) in HCC and PDAC were detected by flow cytometry (*n* = 8). (F) Tumor volume in the orthotopic HCC and PDAC models in different treatment groups (*n* = 10). (G) Representative immunofluorescence images and quantification of TUNEL staining (green) with DAPI counterstain (blue) in tumors after treatment of the orthotopic HCC and PDAC models with different treatments, as described in a (*n* = 7 images from four mice). Scale bar, 10 μm. (H) Number of spontaneously occurring lung metastatic nodules in the orthotopic HCC (HCA-1) model was reduced in mice treated with Dox-cGAMPnp@Gel or aPD1-Dox-cGAMPnp@Gel (*n* = 24). Images of H&E staining of metastatic tumor nodules in the lung. Scale bar, 200 μm. (I) Experimental design to examine the translational potential of the in situ gel vaccine by evaluating STING activation in human tissues. (J) mRNA expression of type I IFN-related genes (*IFNA1* and *IFNB1*) in PDAC patient tumor samples after treatment with cGAMPnp (1 μM) with or without combination of Dox (2 μM) for 6 h. All data are shown as the mean ± SEM *P < 0.05, **P < 0.01, ***P < 0.001, ****P < 0.0001.

To broaden the potential clinical utility of the in situ gel vaccine, we further evaluate its therapeutic efficacy in HCC and PDAC. Given their immunologically “cold” status, these gastrointestinal tumors are notoriously resistant to traditional immunotherapies, posing a significant therapeutic challenge.^30,32^ We first examined changes in the immunosuppressive TME in orthotopic murine HCC (HCA-1) and PDAC (KPC001) models after treatment with the in situ gel vaccine (Figure 8A). After administering the in situ gel vaccine with or without anti-PD-1 antibody in the gel (Dox-cGAMPnp@Gel or aPD1-Dox-cGAMPnp@Gel), we found that the number of tumor-infiltrating DCs did not change in either model (Figure 8B), but there was a significant increase in their activation (CD86^+^MHCII^+^) (Figure 8C). Furthermore, in the HCC model, there was an increase in the number of tumor-infiltrating cytotoxic T cells following administration of Dox-cGAMPnp@Gel or aPD1-Dox-cGAMPnp@Gel (Figure 8D), but the PDAC model did not show a similar increase, likely due to barriers in the desmoplastic stroma. Nonetheless, we noted a significant increase in the activation of these cytotoxic T cells in both models (Figure 8E). Consequently, compared to anti-PD-1 antibody alone (aPD1@Gel) or no treatment, both Dox-cGAMPnp@Gel and aPD1-Dox-cGAMPnp@Gel significantly inhibited primary tumor growth (Figure 8F) and increased the percentage of TUNEL^+^ cells, indicating increased tumor cell apoptosis (Figure 8G) in both models. Because mice bearing orthotopic HCC tumors develop metastases in the lungs 4 weeks after cancer cell implantation, we evaluated the effect of the in situ gel vaccine on metastasis. We observed that both Dox-cGAMPnp@Gel and aPD1-Dox-cGAMPnp@Gel suppressed distal lung metastasis in the HCC model (Figure 8H). In conclusion, the in situ gel vaccine triggered potent anticancer immunity and profoundly impeded tumor progression in these immunologically cold cancers.

To evaluate the translational potential, we explored the feasibility of activating STING using cGAMPnp and Dox in human tissue samples (Figure 8I). We procured freshly resected tumor samples from PDAC patients followed by tissue dissection. Post dissection, the samples were treated with cGAMPnp and Dox for a duration of 6 h. We subsequently employed real-time PCR to detect the mRNA expression of type I IFN-related genes in these treated samples. cGAMPnps moderately upregulated the mRNA expression of *IFNA1* and *IFNB1* (Figure 8J). Moreover, a substantial increase in type I IFN-related gene expression was observed in cells from human PDAC after treatment with combined cGAMPnp and Dox compared with the control treatment (Figure 8J), indicating the translational potential of the in situ gel vaccine. From a safety perspective, we compared hepatic enzyme levels (AST, ALT, and ALP) and renal function indexes (BUN and CREA) between treated and untreated mice and found no significant difference (Table S5), suggesting that the in situ gel vaccine was well tolerated in animal safety tests. With its favorable safety profile and therapeutic efficacy across various malignancies, the in situ gel vaccine expands the scope of immunotherapy, offering significant potential for clinical applications.

## Discussion

Despite extensive efforts to develop delivery systems and materials capable of activating the STING pathway for cancer immunotherapy, significant challenges remain.^49−52^ Our study presents an injectable SF hydrogel-based in situ vaccine for cancer immunotherapy. The codelivery of a nanoscale STING agonist (cGAMPnp), an ICD inducer (Dox), and an immunomodulator (anti-PD-1 antibody or OX40L) in the hydrogel overcome existing limitations such as poor pharmacokinetics and cellular uptake, transient activation, and an immunosuppressive TME that hampered the application of STING agonists. A key aspect of this work is the use of core–shell NPs to efficiently deliver cGAMP, thereby activating the STING pathway and promoting DC maturation. The core–shell structure not only stabilizes the NPs and enhances cellular uptake but also enables controlled cGAMP release, leading to sustained and potent STING activation. In contrast, formulations lacking a core–shell structure, such as cGAMP-liposomes or cGAMP cores without lipid shells, showed only modest improvements in cGAMP uptake, type I IFN production, and DC maturation and were less stable in serum. Additionally, Dox-induced ICD further amplifies and prolongs the effects of cGAMPnps on STING activation and DC maturation, enhancing the efficacy of the antitumor immune response. Collectively, this research introduces a promising immunotherapeutic strategy for treating immunologically “cold” cancers, improving survival rates, and preventing tumor recurrence and metastasis.

Our results highlight the significance of ICD-associated DAMPs in promoting antitumor immunity by modulating STING-mediated immune responses. Previous studies have demonstrated that DAMPs released by dying tumor cells can activate DC maturation, resulting in the presentation of tumor antigens to T cells and the initiation of antitumor immunity.^53^ Building on these results, our findings show that ICD-associated DAMPs can work together with cGAMPnps to augment and extend STING activation and DC maturation, thereby strengthening the antitumor immune response. One possible mechanism for this enhancement is regulation of the intracellular trafficking and lysosomal degradation of cGAMP and STING. Our gene ontology analysis shows ICD-associated DAMPs upregulate vesicle localization pathways involved in STING trafficking or degradation. ICD-associated DAMPs reduce the fusion of cGAMP-puncta with lysosomes, leading to delayed degradation of cGAMP and STING and sustained STING activation.

The underlying mechanisms by which DAMPs influence the fusion of cGAMP puncta with lysosomes require further study. However, one potential mechanism by which DAMPs inhibit the intracellular degradation of cGAMP and STING involves interactions with proteasome-mediated degradation pathways. Specifically, HMGB1 may play a key role, as it binds to and suppresses the promoter of the E3 ligase, thus preventing STING degradation.^54^ We observed that the stability of cGAMP in BMDCs and the expression of type I IFN-related molecules, enhanced by ICD-associated DAMPs, were reduced when HMGB1 was downregulated using siRNA. This suggests that HMGB1, a major DAMP induced by Dox, is crucial for maintaining STING activation. Another possible mechanism is the activation of other inflammatory pathways, such as the Toll-like receptor, RIG-I, STAT, and intracellular calcium regulation pathways, which may enhance STING expression and activation or downstream effectors. These findings provide a foundation for future studies to explore how DAMPs and STING agonists orchestrate a coordinated immune response, suggesting that combining STING agonists with ICD-inducing agents could be a promising cancer immunotherapy strategy.

In this study, we propose a promising strategy to overcome resistance encountered with STING agonist monotherapy. This resistance, often associated with downregulated STING expression, inhibited STING signaling, or upregulation of immunoinhibitory molecules, impedes the clinical translation of STING agonists.^55,56^ Particularly, resistance mechanisms such as the upregulation of immune checkpoints (e.g., PD-1/PD-L1 or indoleamine 2,3-dioxygenase) inherent to 4T1 tumors (Figure S5), along with the induction of T-cell apoptosis and the activation of immunosuppressive regulatory T and B cells, collectively reduce the effectiveness of STING-mediated immunotherapy.^46−48^ The in situ gel vaccine, by integrating cGAMPnps, Dox, and OX40L or anti-PD-1 antibody, simultaneously activates the STING pathway and blocks the immunosuppressive PD-L1 pathway, potentially overcoming resistance mechanisms and thereby amplifying the efficacy of STING activation in cancer therapy. Future studies are encouraged to thoroughly explore the underlying mechanisms by which our gel vaccine negotiates resistance in STING agonist therapies.

Our findings illuminate the role of SF hydrogels as effective vehicles for cancer immunotherapy, owing to their ability for sustained, controlled release of diverse therapeutics.^38^ We showed the enhanced therapeutic potential of an in situ gel vaccine encapsulating an ICD inducer (Dox), a nanoscale STING agonist (cGAMPnps), and immunomodulators (anti-PD-1 antibody or OX40L). The sustained release of these agents ensured extended exposure in the TME, augmenting the therapeutic impact. Specifically, we demonstrated that Dox-loaded SF hydrogels are more toxic to 4T1 breast cancer cells than to BMDCs. This differential effect is likely due to the varying sensitivities of tumor and normal cells to DNA damage and cell cycle arrest caused by Dox. Furthermore, the gel could provide structural support for immune cells, improving their infiltration and retention within the tumor.^57^ When administered into the tumor bed, this in situ gel vaccine significantly induced the STING-driven vascular normalization, reprogrammed the immunosuppressive TME and boosted the quantity and activation of cytotoxic CD3^+^CD8^+^ T cells in tumors, TDLNs and premetastatic lungs.

Our finding highlights the vaccine’s role in T cell activation and their enhanced homing to distal premetastatic niches. Given the pivotal role of metastasis in cancer mortality, targeting the premetastatic niche emerges as a promising strategy to prevent metastasis and improve the prognosis. Potential underpinning mechanisms include the creation of chemokine gradients by activated DCs, systemic immune activation triggering broad T cell mobilization, and antigen spreading from vaccine-induced ICD, facilitating T-cell-mediated recognition and elimination of metastatic cells.^58^ Further understanding of these processes in the future studies may bolster the antimetastatic efficacy of this approach.

This study reports a significant advancement of STING-based immunotherapy with marked potential for clinical translation. One promising finding of this study is the demonstration of STING activation in patient-derived PDAC samples after treatment with combined cGAMPnps and Dox, suggesting the translational potential of the in situ gel vaccine. While our findings must be further validated and optimized via clinical trials, they symbolize an important stride in the field. This hydrogel-based, multiagent approach could potentially revolutionize cancer immunotherapy, offering a personalized, effective strategy that broadens the clinical application of STING agonists.

## Conclusions

In conclusion, this study demonstrates the potential of an SF hydrogel-based in situ cancer vaccine, encapsulating cGAMP-loaded NPs (cGAMPnps), ICD inducers, and immunomodulators, to achieve robust and sustained STING activation for cancer immunotherapy. This approach addresses key limitations of STING agonists, such as poor pharmacokinetics and transient activation, by enabling a controlled and prolonged release of therapeutic agents. The combination of STING activation, ICD, and immunomodulation effectively reprograms the TME, enhances DC and CD8^+^ T cell activation, and suppresses tumor progression and metastasis in multiple tumor models, including immunologically cold tumors like glioblastoma and PDAC. This hydrogel-based strategy shows great potential for improving clinical outcomes in cancer immunotherapy and requires further exploration in translational studies.

## Methods

Additional materials and methods are included in Supporting Information (see materials and methods).

### Preparation and Characterization of cGAMPnp

Liposomes composed of DOTAP, DOPC, and cholesterol (in a 1:1:2 molar ratio) were prepared by thin-film hydration, followed by 36 cycles of sonication for a total of 3 min on ice. Each cycle included a 5 s sonication pulse followed by a 5 s pulse-off period (at 40 W power), using a Q125 sonicator (Qsonica LLC). To prepare the core of the NPs, calf thymus DNA (24 μg) and cGAMP (18 μg) were dissolved in 30 μL of deionized water, forming a DNA-cGAMP mixture. Protamine (16 μg) was dissolved in another 30 μL of deionized water and was mixed with the DNA-cGAMP mixture and then allowed to stand at room temperature (RT) for 10 min. The DNA-cGAMP-protamine cores were subsequently mixed with 30 μL of liposomes and left to stand at RT for another 10 min. The size and surface charge of the cGAMPnps were determined at RT using a Zetasizer Pro Blue Label instrument (Malvern Panalytical, UK). The cGAMPnps were resuspended in deionized water, and their characteristics were assessed using the Zetasizer, with a lipid concentration at 0.5 mM. For surface charge measurement, the cGAMP NPs (cGAMPnps) were resuspended in PBS (phosphate buffered saline) (pH 7.4). TEM (H-7500, Hitachi High-Tech) was used to examine the size and morphology of the cGAMPnp.

### Preparation and Characterization of SF Hyrogel

For gel formation, the SF solution was sonicated using a Q125 sonicator (Qsonica LLC) for 100 s with 5 s pulses of sonication, followed by a 5 s pulse-off period (power: 30 W). The solution was then incubated at 37 °C in a dry bath incubator to allow for gel formation. For the incorporation of immunotherapeutic agents, we mixed SF with solutions of Dox, cGAMPnps, anti-PD1 Ab, or OX40L in a volume ratio of 3:1. To analyze the structure of the silk hydrogel, the silk solution was sonicated as described above, followed by freeze-drying with a lyophilizer. Finally, an SEM image was taken.

### Animals and Orthotopic TNBC, HCC, PDAC, and GBM Models

BALB/cByJNarl female mice, C57BL/6JNarl female mice, C3H/HeNCrNarl male mice, and C57BL/6JNarl male mice were purchased from the National Laboratory Animal Center (Taipei, Taiwan) and BioLASCO Taiwan Company (Taipei, Taiwan). 4T1 cells were orthotopically implanted into the mammary fat pad of 6–8 week-old female BALB/cByJNarl mice. Murine HCC HCA-1 cells were orthotopically implanted in the livers of 6–8 week-old male C3H mice. KPC001 cells were orthotopically implanted into the pancreas of 5–6 week-old female C57 mice. ALTS1C1 cells were intracranially (i.c.) injected at 0.1 mm posterior to the bregma and 2.0 mm lateral to the midline with a depth of 2.5 mm depth. The injection hole was then sealed with bone wax (W810, ETHICON), followed by two stitches to suture the skin of the mice. All animals received humane care in compliance with the “Guide for the Care and Use of Laboratory Animals” published by the National Academy of Sciences, and all study procedures and protocols were approved by the Animal Research Committee of National Tsing-Hua University.

### Statistical Analyses

Statistical analyses were performed using GraphPad Prism 9. Student’s *t* tests or Mann–Whitney U-tests were used for comparison between two groups according to data distribution. One-way analysis of variance followed by the Tukey post hoc test was used for the comparison of three or more groups. Values were normally distributed, and the variance was similar between compared groups. Comparison of survival curves was performed using a log-rank Mantel–Cox test (two-sided). *P* < 0.05 was considered statistically significant.

## Data Availability

All data relevant to the study are included in the article or uploaded as Supporting Information.

## Supporting Information Available

Information on gene ID, fold change, gene type, and gene name (XLSX)
Materials and methods; size and PDI of core–shell cGAMPnp, cGAMP in liposomes without a core–shell structure (cGAMP-liposome), and cGAMP-DNA-protamine cores without lipid shells (cGAMP-core) after 2 h of incubation with 20% FBS in PBS; HALLMARK pathway sets revealed differential presence of cell signaling pathways in human breast cancer samples treated with DOX relative to untreated control; KEGG pathway sets revealed higher expression of inflammatory and STING activation regulatory pathways in human breast cancer samples treated with DOX relative to untreated control; GOBP pathway sets revealed differential presence of inflammatory and STING activation regulatory pathways in BMDC among groups relative to Control; in vivo administration of the in situ gel vaccine (Dox-cGAMPnp@Gel) did not show systemic toxicity; fluorescein-cGAMP uptake by BMDCs treated with cGAMPnps, cGAMP-liposome or cGAMP core; the mRNA expressions of type I IFN and inflammation-related genes in BMDCs 48 h after treatment with cGAMPnp, ICD-associated DAMPs, or the combination of both were measured by RT–qPCR; effect of HMGB1 downregulation on the stability of cGAMP and expression of type I IFN-related molecules in BMDCs; viability of BMDCs and 4T1 cells after 24 h exposure to blank or Dox@Gel; PD-L1 expression in 4T1 TNBC cells and immune profiling in tumor-bearing mice; gating strategy for flow cytometry to determine T cell and DC subsets in lymph nodes (PDF)

## Abbreviations

DCs: dendritic cells
ICD: immunogenic cell death
DAMPs: damage-associated molecular patterns
cGAS: cyclic GMP–AMP synthase
STING: stimulator of interferon genes
cGAMP: cyclic GMP-AMP
IFN: interferon
NK cells: nature killer cells
SF: silk fibroin
cGAMPnps: cGAMP-loaded nanoparticles
DOTAP: 1,2-dioleoyl-3-trimethylammonium-propane
TME: tumor microenvironment
TNBC: triple-negative breast cancer
GBM: glioblastoma
HCC: hepatocellular carcinoma
PDAC: pancreatic ductal adenocarcinoma
NPs: nanoparticles
TEM: transmission electron microscopy
BMDCs: bone marrow-derived dendritic cells
TBK1: TANK-binding kinase 1
IRF3: interferon regulatory factor 3
MHC-II: major histocompatibility complex class II
SEM: scanning electron microscopy
Dox@Gel: Dox-loaded SF hydrogels
cGAMPnp@Gel: cGAMPnp-loaded SF hydrogels
Dox-cGAMPnp@Gel: SF hydrogels loaded with both Dox and cGAMPnps
TDLNs: tumor-draining lymph nodes
aPD1-Dox-cGAMPnp@Gel: SF hydrogels loaded with anti-PD-1Dox and cGAMPnps
OX40L-Dox-cGAMPnp@Gel: SF hydrogels loaded with OX40LDox and cGAMPnps
aPD1@Gel: anti-PD-1-loaded SF hydrogels
OX40L@Gel: OX40-L-loaded SF hydrogels
TUNEL: terminal deoxynucleotidyl transferase dUTP nick end labeling
AST: aspartate transaminase
ALT: alanine transaminase
ALP: alkaline phosphatase
BUN: blood urea nitrogen
CREA: creatinine
H&E: hematoxylin and eosin
