# Supplementary material for: Robust and Sustained STING Pathway Activation via Hydrogel-Based In Situ Vaccination for Cancer Immunotherapy
Source: ACS Nano. 2024 Oct 15;18(43):29439–56. doi: 10.1021/acsnano.3c12337 (PMC11526424; doi:10.1021/acsnano.3c12337)
Supplement: Supplementary file 2 — nn3c12337_si_002.pdf [file nn3c12337_si_002.pdf]

Supporting Information

Robust and Sustained STING Pathway Activation via Hydrogel-Based In-Situ Vaccination for Cancer Immunotherapy

Sheng-Liang Cheng[1,2,#], Hsin-Mei Lee[1,#], Chung-Pin Li[3,4,5,6#], Mei-Wei Lin[7], Min-Yuan Chou[7], Yu-Ting Yen[8], Tun-Han Wu[1], Yun-Chen Lian[1], Yu-Chuan Shih[1], Chi-Shiun Chiang[9], Ting-Wen Chen[10,11,12], Dehui Wan[1,]*, Yunching Chen[1,13]**

[1] Institute of Biomedical Engineering, National Tsing Hua University, Hsinchu 30013, Taiwan

[2] International Intercollegiate Ph.D. Program, National Tsing Hua University, Hsinchu 30013, Taiwan

[3] Division of Gastroenterology and Hepatology, Department of Medicine, Taipei Veterans General Hospital, Taipei 11217, Taiwan

[4] Division of Clinical Skills Training, Department of Medical Education, Taipei Veterans General Hospital, Taipei 11217, Taiwan

[5] Therapeutic and Research Center of Pancreatic Cancer, Veterans General Hospital, Taipei 11217, Taiwan

[6] School of Medicine, College of Medicine, National Yang Ming Chiao Tung University, Taipei 112304, Taiwan

[7] Biomedical Technology and Device Research Laboratories, Industrial Technology Research Institute, Hsinchu 310401, Taiwan.

[8] Institute of Translational Medicine and New Drug Development, School of Medicine, China Medical University, Taichung 406040, Taiwan

[9] Department of Biomedical Engineering and Environmental Sciences, National Tsing Hua University, Hsinchu 30013, Taiwan

[10] Institute of Bioinformatics and Systems Biology, National Yang Ming Chiao Tung University, Hsinchu, 30068, Taiwan

[11] Department of Biological Science and Technology, National Yang Ming Chiao Tung University, Hsinchu, 30068, Taiwan

[12] Center for Intelligent Drug Systems and Smart Bio-devices, National Yang Ming Chiao Tung University, Hsinchu 30068, Taiwan

[13] Department of Chemistry, National Tsing Hua University, Hsinchu 30013, Taiwan

[#] These authors contributed equally: *Sheng-Liang Cheng, Hsin-Mei Lee, Chung-Pin Li*

Supplementary Method and Materials

Gene set enrichment analysis of single-cell gene expression database of human breast cancer samples

A publicly available single-cell gene expression database of human breast cancer samples treated with doxorubicin was analyzed to demonstrate alterations in gene expression. For pre-processing of RNA-seq Datasets, the raw counts were normalized for gene length and transformed to TPM (transcripts per million) values, which were then log2 normalized to obtain the final values. For gene set enrichment analysis, total gene analysis was performed using Hallmark Gene Set. Ranked gene set enrichment analysis (GSEA) was performed on the full ranked gene lists.[1] Furthermore, selected genes with high expression in DOX treated group were performed KOBAS.[2]

Cells and materials

Protamine sulfate salt (fraction X from salmon) and calf thymus DNA were purchased from Sigma Aldrich (St. Louis, MO). 1,2-Dioleoyl-3-trimethylammonium-propane (DOTAP), 1,2-Dioleoyl-sn-glycero-3-phosphocholine (DOPC), and cholesterol were purchased from Avanti Polar Lipids (Alabama, USA), Inc. 2'3'-cGAMP was purchased from Invivogen (California, USA), and c[8-Fluo-AET-G(2',5’) pA(3',5')p] (fluorescein-cGAMP) was purchased from Biolog Life Science Institute (Bremen, Germany). FITC-conjugated albumin from bovine serum (FITC-BSA) was purchased from Thermo Fisher Scientific (USA), and Doxorubicin was purchased from LC Laboratory (Massachusetts, USA).

The murine TNBC 4T1 cells were purchased from ATCC (no. ATCC CRL-2539™). The murine HCC cell line HCA-1 was kindly provided by D. Duda from Massachusetts General Hospital (Boston, USA). The murine PDAC KPC001 cells (KrasG12Dp53R172H/+) were kindly provided by Dr. Yves Boucher, from Massachusetts General Hospital (Boston, USA), and were isolated from mice with spontaneous pancreatic tumors (Pdx1Cre/LSLKrasG12D/p53R172H/+).[3] The murine astrocytoma cell line ALTS1C1 was purchased from the Bioresource Collection and Research

Center (Hsinchu, Taiwan). 4T1, HCA-1, KPC001, and ALTS1C1 cells were all cultured in high-glucose Dulbecco's modified Eagle's medium (DMEM). All media were supplemented with 10% FBS (HyClone, Logan, UT) and 1% penicillin and streptomycin antibiotics (HyClone, Logan, UT). All cells were cultured at 37°C in a Forma 370 incubator (Thermo Fisher Scientific, USA) with an atmosphere of 5% CO2.

BMDCs were generated using the following procedure: First, the femur and tibia were harvested from BALB/cByJNarl female mice. The bone marrow cavities were then flushed with Roswell Park Memorial Institute (RPMI)-1640 medium and differentiated into BMDCs at a density of 2×10^5 cells/mL in RPMI-1640 medium. This medium was supplemented with 10% heat-inactivated FBS (HyClone, Logan, UT), 1% penicillin and streptomycin antibiotics (HyClone, Logan, UT), 20 ng/mL Granulocyte-macrophage colony-stimulating factor (GM-CSF, R&D systems, MN, USA), and 0.05 mM β-ME (Thermo Fisher Scientific, USA). The cells were incubated at 37°C in an atmosphere of 5% CO2. On the third day, an additional 5 mL of RPMI complete medium with 20 ng/mL GM-CSF was added. Finally, the BMDCs were harvested on the sixth day and used for further experiments.

Construction and expression of murine OX40 ligand collabody

For the construction of a hexavalent OX40 ligand collabody, the cDNA (OX40L2) coding for the extracellular domain of murine OX40 ligand was fused to both the N and C termini of a collagen-like peptide, $(\text{Gly-Pro-Pro})_{10}$. This peptide is capable of trimerizing the fusion partners when expressed in mammalian cells. The expression construct, pLVU2-OX40L2_Hygro, was transfected into CHO-S cells by electroporation and followed by hygromycin selection. The fed-batch culture of OX40L collabody-expressing CHO-S clones was performed in Acti-Pro medium with chemically-defined supplements and glucose in shaking flasks. The secreted OX40L collabody was purified using Strep-Tactin™ XT Superflow™ High Capacity Resin (IBA Lifesciences, Goettingen, Germany). The activity of OX40L was measured and monitored using an OX40 Bioassay (Promega, # JA4191).

Release of cGAMP from the cGAMPnp

To measure the release of cGAMP from the cGAMPnp, the FAM-labeled cGAMP (fluorescein-cGAMP) was used to formulate the fluorescein-cGAMPnps. The release properties of the fluorescein-cGAMPnps were then assessed in conditions of pH 7.4 PBS buffer or pH 4 acetic acid buffer at 37°C. Finally, the release rates were calculated from the fluorescence intensity of fluorescein-cGAMP (excitation at 494 nm, emission at 517 nm) measured at different time points using a plate reader (Spark 10M, Tecan).

Cellular uptake of cGAMP

BMDCs treated with either free fluorescein-cGAMP or fluorescein-cGAMPnp were fixed in acetone at -20 °C for 10 minutes and then washed with PBS. The sections were subsequently stained with DAPI (Vector Laboratories, California, USA) and imaged using a confocal laser scanning microscope (LSM780, Zeiss). Images were quantified using random fields per group. The expression level was quantified by measuring the area occupied by the staining of interest, normalized by the area of DAPI-stained nuclei. This was expressed as the ratio of green to blue (FITC/DAPI) relative fluorescence units. The fluorescence intensity was analyzed using ImageJ.

Western blot analysis

Cells were lysed in RIPA lysis buffer for 30 minutes on ice following treatment. The supernatant was collected after centrifugation at 12,000 rcf and mixed with 4× Laemmli Sample buffer (Bio-Rad, California, USA). Cell lysates were separated on a 10% acrylamide gel and transferred to a PVDF membrane. Membranes were blocked for 1 hour with 5% BSA in TBST and then incubated overnight with primary antibodies against STING (D2P2F) (no. 13647, Cell Signaling Technologies, Massachusetts, USA), phospho-TBK1 (Ser172) (D52C2) (no. 5483, Cell Signaling Technologies, Massachusetts, USA), TBK1 (D1B4) (no. 3504, Cell Signaling Technologies, Massachusetts, USA), phospho-IRF3 (Ser396) (D6O1M) (no. 29047, Cell Signaling Technologies,

Massachusetts, USA), IRF3 (D83B9) (no. 4303, Cell Signaling Technologies) and β-actin (no. A5441, Sigma Aldrich, Missouri, USA).

RNA sequencing

The RNA sequencing library was prepared using ureSelect XT HS2 mRNA Library Preparation Kit (Agilent, USA) and the sequencing was performed using Illumina NovaSeq 6000 platform to generate 150 bp paired-end reads. Raw FASTQ files were trimmed by Trimmomatic to remove bases with quality lower than 30. Expression levels were estimated with RSEM and mouse genome and annotation downloaded from GENCODE were used as reference [4, 5]. Significant DEGs (differentially expressed genes) were identified with DESeqV2 and genes with absolute fold change larger than 2 and adjusted p value less than 0.05 were defined as DEGs [6]. R package, clusterProfiler were used to perform GSEA analysis with biological process pathways from GO database [1, 7-9]. Heatmaps were generated for the z-transformed normalized TPM expression levels derived from RSEM with R package ComplexHeatmaps [10]. Volcano plot were generated from the adjusted p valued and fold changes derived from DESeq2 with R package ggplot2 [11].

Reverse transcription-quantitative polymerase chain reaction (RT-qPCR)

Total RNA was extracted from BMDCs, tumor tissues, or lymph nodes using TRIzol reagent (Life Technologies, California, USA). The cDNAs were synthesized using the High-Capacity cDNA Reverse Transcription Kit (Applied Biosystems, Massachusetts, USA). We used primers specific for *ifna, ifnb, cxcl10, tnfa, actb, IFNB, IFNA*, and *ACTB*, and determined the relative gene expression with a PowerUp SYBR Master Mix (Applied Biosystems, Massachusetts, USA) on a QuantStudio™ 3 Real-Time PCR System (Applied Biosystems, Massachusetts, USA). The comparative threshold cycle method was utilized to calculate the fold change in gene expression, which was normalized to Actb or ACTB as reference genes. The primer sequences used were as follows:

Genes	Forward primers (5'→3')	Reverse primers (5'→3')
mifnb	GGAAAGATTGACGTGGGAGA	CCTTTGCACCCTCCAGTAAT
mifnα	TGTCTGATGCAGCAGGTGG	AAGACAGGGCTCTCCAGAC
mcxcl10	CCAAGTGCTGCCGTCATTTTC	GGCTCGCAGGGATGATTTCAA
mtnfα	CCGATGGGTTGTACCTTGT	CGGACTCCGCAAAGTCTAAG
mactb	TGAGAGGGAAATCGTGCGTG	TTGCTGATCCACATCTGCTGG
hIFNB	GTTCGTGTTGTCAACATGACCA	TCAATTGCCACAGGAGCTTCT
hIFNA	GCCTCGCCCTTTGCTTTACT	CTGTGGGTCTCAGGGAGATCA
hACTB	CATGTACGTTGCTATCCAGGC	CTCCTTAATGTCACGCACGAT

ELISA

The cell supernatant from the BMDCs treated with different formulations was collected to detect the production of IFN-β and CXCL10. Detection was carried out using the mouse IFN-β and CXCL10 ELISA kit according to the manufacturer's instructions (BD Biosciences, California, USA).

Flow cytometry analysis

To assess DC maturation *in vitro*, BMDCs treated with various formulations were subjected to staining with CD45-FITC, CD11c-APC, CD86-PE-Cy7, and MHC II-APC-eflour780. The activation of DCs was determined by analyzing the co-expression of CD86 and MHC II, markers associated with DC activation, within the CD45+/CD11c+ DC population.

To analyze the immune cell population *in vivo*, mice were perfused with PBS via intra-cardiac injection and then euthanized. Tumor, lymph nodes, and lung tissues were digested at 37°C for 1 hour using collagenase type 1A (1.5 mg/ml) and hyaluronidase (1.5 mg/ml) in DMEM medium. Subsequently, cell suspensions were stained for further analysis. The following antibodies were used for flow cytometry analysis: CD45-FITC (no. 30-F11), CD3e-APC (no. 145-2C11), CD8-PE-Cy7(no. 53-6.7), CD4-PE (no. RM4-5), CD11c-APC (no. 550261), IFN-γ-APC-Cy7 (no. 561479), CD86-PE-Cy7 (no. 560582), CD44-PE (no. 561860), FITC-CD62L (no. 561917), CD16/CD32 BD Fc Block (no. 2.4G2), and 7-AAD, all from BD Biosciences (California, USA); MHC

II-APC-eflour780 (no. 47-5321-82) from eBioscience (California, USA). Flow cytometry data were obtained from a BD FACSAria III flow cytometer (Becton Dickinson) and analyzed with FACSDiva™ software. The gating strategy was shown in **Figure S6**.

To detect IFN-γ in CD8 T cells, cell suspensions were fixed in a 4% paraformaldehyde solution, permeabilized in Cytofix/Cytoperm solution (BD Biosciences, California, USA), and stained with an intracellular IFN-γ-APC-Cy7 (BD Biosciences, California, USA) according to the manufacturer's instructions.

Preparation of silk fibroin (SF)

To prepare the silk fibroin, cocoons were initially heated in boiling DI water with 0.02M Na2CO3 for 30 minutes to remove the sericin. The fibroin was then rinsed three times with DI water and dried overnight in an incubator. Subsequently, a 9.3M LiBr solution was prepared and added to dissolve the silk fibroin at 60°C for 4 hours. The resulting solution was dialyzed in a dialysis bag for 48 hours. To remove debris, the silk fibroin solution was centrifuged twice at 9000rpm (at 4°C for 20 minutes).

Encapsulation efficiency and release profile of therapeutic cargoes in hydrogels

The encapsulation efficiency of various therapeutic cargoes in hydrogels was evaluated using Dox, fluorescein-cGAMPnp, Alexa-Fluor488-labeled PD1 Ab, and Alexa-Fluor488-labeled OX40L. The fluorescence intensities of Dox (excitation at 470 nm, emission at 595 nm), fluorescein-cGAMPnp, Alexa-Fluor488-labeled PD1 Ab, and Alexa-Fluor488-labeled OX40L (excitation at 494 nm, emission at 517 nm) were measured using a plate reader (Spark 10M, Tecan).

The release profiles of different therapeutic cargoes from the hydrogels were assessed in pH 7 PBS buffer or pH 6.8 acetic acid buffer at 37°C, with the encapsulation of Dox, fluorescein-cGAMPnp, or FITC-BSA, respectively. The fluorescence intensities of Dox (excitation at 470 nm, emission at 595 nm), fluorescein-cGAMPnp, and FITC-BSA (excitation at 494 nm, emission at 517 nm) were measured using a plate reader (Spark 10M, Tecan). The release of Dox, fluorescein-

cGAMPnp, and FITC-BSA from the hydrogels *in vivo* was evaluated using an *in vivo* imaging system (IVIS, PerkinElmer). Mice were anesthetized to measure the fluorescence intensities of Dox, fluorescein-cGAMPnp, or FITC-BSA in the tumors at different time points.

Immunostaining

Tumor tissues were first fixed with paraformaldehyde (PFA in PBS) for 8 hours and incubated in 30% sucrose solution overnight. Next, the fixed tissues were embedded in Tissue-Tek (OCT compound) and kept frozen at −80 °C. Each tissue was then sectioned with a thickness of 10 μm on a slide. The frozen sections were fixed in acetone at −20 °C for 10 min and washed with PBS. Subsequently, the sections were blocked with 5% bovine serum albumin solution for 1 h at room temperature and incubated with primary antibodies against CD31 (no. ab28364, Abcam) and NG2 (clone no. 132.38, Millipore) at 4 °C overnight. Sections were then washed with PBS and incubated with either Alexa Fluor 488 anti-rabbit IgG or Dylight 594 anti-mouse IgG secondary antibodies (Thermo Scientific) for 1 h at room temperature. Finally, Sections were washed by PBS and counterstained with DAPI (Vector Laboratories, Burlingame, CA). All sections were imaged by a confocal laser scanning microscope (LSM 780, Zeisss, Germany).

Tumor perfusion

Nine days after the orthotopic 4T1 tumor cell implantation, the mice were treated with empty Gel, Dox@Gel, cGAMPnp@Gel, Dox-cGAMPnp@Gel or Free Dox-cGAMPnp on days 9, 16 and 23; 200 μg of Hoechst 33342 was injected intravenously into the mice 5 min before sacrifice on day 30, after which the tumors were harvested. The Hoechst 33342-positive cells in different treatment groups were then calculated by the BD FACSAria III flow cytometer (Becton Dickinson) and analyzed with FACSDiva™ software.

Assessment of apoptosis by terminal deoxynucleotidyl transferase dUTP nick end labeling (TUNEL) staining

Frozen sections of tumors were stained using the DeadEnd™ Fluorometric TUNEL System (G3250, Promega) according to the manufacturer's recommendations. Apoptotic cells were counted in randomly selected visual fields for each treatment group. The apoptotic index was calculated as the fraction of apoptotic nuclei.

H&E staining

Tissues collected from different treatment groups were first diced into small pieces and then fixed in 4% paraformaldehyde overnight. The fixed tissue was embedded in paraffin wax, and sections were prepared for further staining with hematoxylin and eosin. Finally, the stained sections were observed through a Nikon microscope (Eclipse E800).

Calculation of drug synergism

The drug synergism was calculated by Bliss Independence model which is one of the effect-based strategies for the evaluation of drug synergism. The expected combined effect between two drugs was defined as: $E_{AB} = E_A + E_B(1 - E_A)$, where E_A represent the effect of drug A, E_B represent the effects of drug B, and E_{AB} was the effect of drug A combined with drug B. The calculation index (CI) could then be shown as $CI = \frac{E_A + E_{B-E_A E_B}}{E_{AB}}$ where the synergism happen between two drugs when CI value over 1[12] .

Table S1. Size and PDI of core-shell cGAMPnp, cGAMP in liposomes without a core-shell structure (cGAMP-liposome), and cGAMP-DNA-protamine cores without lipid shells (cGAMP-core) after 2 hours of incubation with 20% FBS in PBS.

Sample	Size (nm)	PDI
cGAMP-core	85 ± 10	0.801 ± 0.172
cGAMP-liposome	3534 ± 1407	0.914 ± 0.150
cGAMPnp	188 ± 2	0.248 ± 0.011

Table S2. HALLMARK pathway sets revealed differential presence of cell signaling pathways in human breast cancer samples treated with DOX relative to untreated control.

**Name of gene set**	**ES**	**NES**	**adjusted P value**
Estrogen response early	194	0.41	0
**Interferon-gamma response**	**182**	**0.41**	**0.001**
Epithelial mesenchymal transition	182	0.35	0.011
Estrogen response late	193	0.35	0.017
**Interferon-alpha response**	**93**	**0.38**	**0.037**
KRAS signaling dn	157	0.34	0.037
Complement	179	0.33	0.045

Table S3. KEGG pathway sets revealed higher expression of inflammatory and STING activation regulatory pathways in human breast cancer samples treated with DOX relative to untreated control.

Name of gene set	Input	Background	P-Value
**Innate Immune System**	89	1043	1.91E-28
**Adaptive Immune System**	56	748	2.5E-14
**Interleukin-1 signaling**	20	101	5.86E-12
**Class I MHC mediated antigen processing & presentation**	35	370	8.75E-12
**Cytokine Signaling in Immune system**	54	836	1.25E-11
**FCERI mediated NF-kB activation**	16	81	9.48E-10
**MHC class II antigen presentation**	12	123	0.0001
**Toll Like Receptor 3 (TLR3) Cascade**	9	93	0.0008
**Antigen processing and presentation**	8	77	0.001
**MyD88-independent TLR4 cascade**	9	98	0.001
**Regulation of innate immune responses to cytosolic DNA**	4	15	0.002
**Toll-like Receptor Cascades**	11	154	0.002
**STING mediated induction of host immune responses**	3	16	0.015

Table S4. GOBP pathway sets revealed differential presence of inflammatory and STING activation regulatory pathways in BMDC among groups relative to Control.

A. Treatment of cGAMP NPs Vs Control

Name of gene set	ES	NES	adjusted P value
regulation of inflammatory response	0.325	1.283	0.060
cellular response to type I interferon	0.627	1.866	0.009
cellular response to interferon-alpha	0.897	2.097	0.007
cellular response to interferon-beta	0.911	2.787	0.007
cellular response to interferon-gamma	0.728	2.566	0.007
cellular response to tumor necrosis factor	0.519	1.876	0.007
interleukin-1 production	0.459	1.589	0.018
toll-like receptor signaling pathway	0.536	1.882	0.007
MyD88-dependent toll-like receptor signaling pathway	0.561	1.428	0.131
RIG-I signaling pathway	0.703	1.767	0.020
I-kappaB kinase/NF-kappaB signaling	0.313	1.177	0.176
STAT cascade	0.469	1.684	0.010
ERK1 and ERK2 cascade	0.379	1.484	0.008
regulation of cytosolic calcium ion concentration	0.289	1.137	0.232
receptor-mediated endocytosis	0.279	1.065	0.330
vesicle localization	0.313	1.219	0.127
lysosome organization	0.354	1.152	0.243
blood vessel remodeling	0.645	1.433	0.134

B. Treatment of conditioned medium from Dox-treated 4T1 cells Vs Control

Name of gene set	ES	NES	adjusted P value
regulation of inflammatory response	0.336	1.390	0.015
cellular response to type I interferon	0.592	1.781	0.007
cellular response to interferon-alpha	0.808	1.891	0.005
cellular response to interferon-beta	0.843	2.641	0.005
cellular response to interferon-gamma	0.594	2.137	0.005
cellular response to tumor necrosis factor	0.471	1.741	0.005
interleukin-1 production	0.448	1.590	0.007
toll-like receptor signaling pathway	0.412	1.482	0.022
MyD88-dependent toll-like receptor signaling pathway	0.553	1.446	0.076
RIG-I signaling pathway	0.570	1.478	0.070
I-kappaB kinase/NF-kappaB signaling	0.331	1.292	0.068
STAT cascade	0.384	1.412	0.024
ERK1 and ERK2 cascade	0.439	1.788	0.005
regulation of cytosolic calcium ion concentration	0.423	1.733	0.005
receptor-mediated endocytosis	0.432	1.696	0.005
vesicle localization	0.316	1.269	0.067
lysosome organization	0.433	1.440	0.044
blood vessel remodeling	0.739	1.611	0.024

C. Treatment of combined cGAMP NPs and conditioned medium from Dox-treated 4T1 cells Vs Control

Name of gene set	ES	NES	adjusted P value
regulation of inflammatory response	0.355	1.458	0.009
cellular response to type I interferon	0.642	1.913	0.004
cellular response to interferon-alpha	0.874	2.014	0.004
cellular response to interferon-beta	0.915	2.826	0.004
cellular response to interferon-gamma	0.743	2.654	0.004
cellular response to tumor necrosis factor	0.522	1.920	0.004
interleukin-1 production	0.489	1.719	0.004
toll-like receptor signaling pathway	0.509	1.812	0.004
MyD88-dependent toll-like receptor signaling pathway	0.668	1.726	0.015
RIG-I signaling pathway	0.670	1.718	0.015
I-kappaB kinase/NF-kappaB signaling	0.376	1.468	0.015
STAT cascade	0.466	1.698	0.004
ERK1 and ERK2 cascade	0.417	1.695	0.004
regulation of cytosolic calcium ion concentration	0.363	1.490	0.005
receptor-mediated endocytosis	0.401	1.579	0.005
vesicle localization	0.316	1.273	0.056
lysosome organization	0.434	1.400	0.056
blood vessel remodeling	0.759	1.633	0.020

Table S5. ***In vivo*** **administration of the in situ gel vaccine (Dox-cGAMPnp@Gel) did not show systemic toxicity.** Measurement of the levels of hepatic and renal toxicity markers in serum of healthy Balb/c mice with Dox-cGAMPnp@Gel (0.8 mg/kg cGAMP and 2.0 mg/kg Dox). Serum samples were taken 24 h or 48 h after treatment (n=4). *Abbreviations:* ALT, alanine aminotransferase; AST, aspartate aminotransferase; ALP, alkaline phosphatase; BUN, blood urea nitrogen; CREA, creatinine. Data are shown as the mean values ± SD.

Treatment	AST (U/L)	ALT (U/L)	ALP (U/L)	BUN (mg/dL)	CREA (mg/dL)
**Control**	59.6±3.54	22.8±0.81	290.6±6.87	26.0±0.36	0.16±0.003
**24h after injection**	77.5±3.97	31.2±0.86	297.1±4.59	30.8±1.54	0.19±0.008
**48h after injection**	59.6±2.79	27.5±0.81	250.1±7.94	23.6±1.01	0.18±0.008

Figure S1. Fluorescein-cGAMP uptake by BMDCs treated with cGAMPnps, cGAMP-liposome or cGAMP core. The cellular uptake of fluorescein-cGAMP (1 μM) was imaged and quantified using a Zeiss LSM 780 confocal microscope (n=6). All data are shown as the mean ± SEM. **P*<0.05, ***P*<0.01, ****P*<0.001.

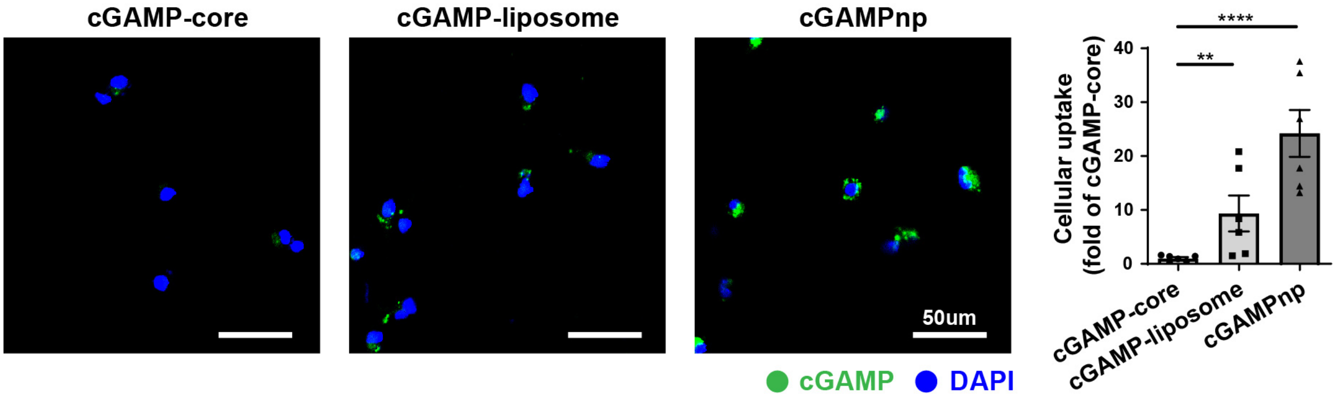

Figure S2. The mRNA expressions of type I IFN and inflammation-related genes (IFNB1 AND IFNA1) in BMDCs 48h after treatment with cGAMPnp (1 μM), ICD-associated DAMPs (conditioned medium collected from oxaliplatin-treated 4T1 cells), or the combination of both were measured by RT–qPCR. The results are expressed as the fold change relative to the corresponding level in the untreated control group (n=3). All data are shown as the mean ± SEM. *P<0.05, **P<0.01, ***P<0.001.

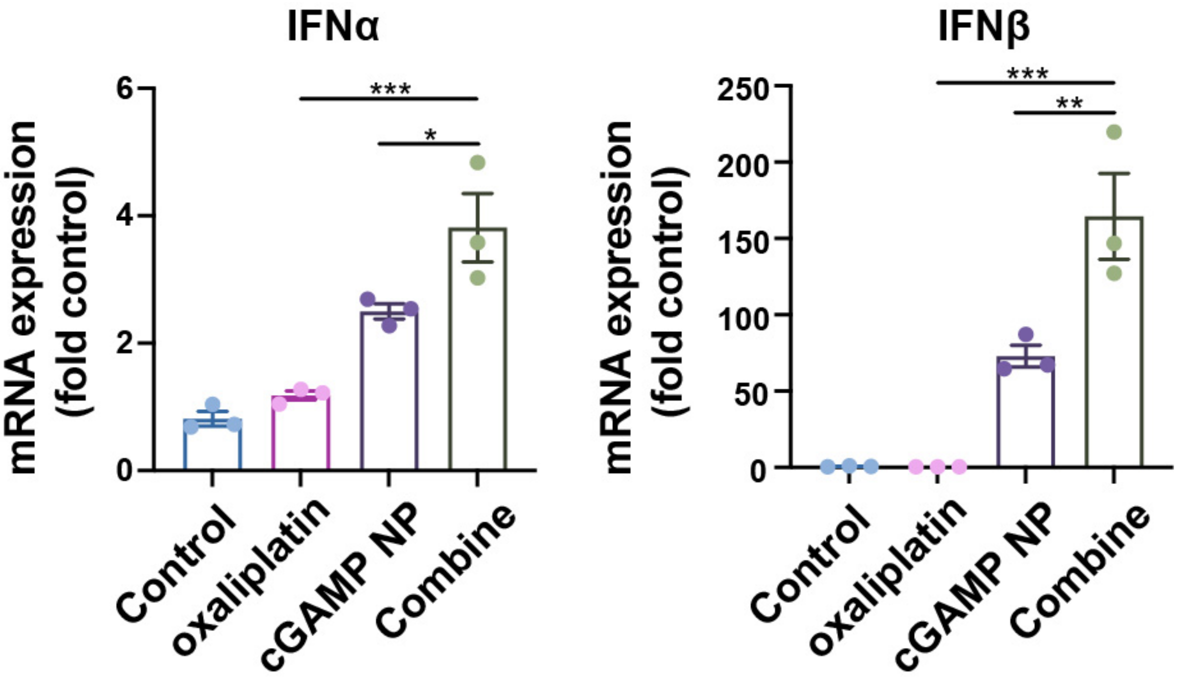

Figure S3. Effect of HMGB1 downregulation on the stability of cGAMP and expression of type I IFN-related molecules in BMDCs. (**A**) Schematic Representation of Experimental Design. 4T1 cells transfected with either control siRNA or HMGB1 siRNA were treated with Dox (2 μM) for 6 hours. The medium was then replaced with fresh medium, and the conditioned medium was collected after 24 hours. BMDCs were treated with cGAMPnps (1 μM) and the collected conditioned medium. (**B**) BMDCs were treated with cGAMPnps (green) in combination with ICD-associated DAMPs. The conditioned medium from 4T1 breast cancer cells transfected with control or HMGB1 siRNA and exposed to Dox for 24 hours was used to treat BMDCs, which were then incubated with cGAMPnps loaded with fluorescein-cGAMP for 12 hours. Scale bar, 10 μm. (**C**) The mRNA expressions of type I IFN and inflammation-related genes in BMDCs were measured 24 hours after treatment with cGAMPnp (1 μM) and ICD-associated DAMPs from Dox-treated 4T1 cells transfected with control or HMGB1 siRNA. Results are expressed as the fold change relative to the corresponding level in BMDCs treated with cGAMPnp and ICD-associated DAMPs from Dox-treated 4T1 cells (n=4). (**D**) IFN-β production in BMDCs was measured by ELISA 24 hours after treatment with cGAMPnp (1 μM) and ICD-associated DAMPs from Dox-treated 4T1 cells transfected with control siRNA or HMGB1 siRNA (n=4). Data are shown as the mean ± SEM. ***P<0.001.

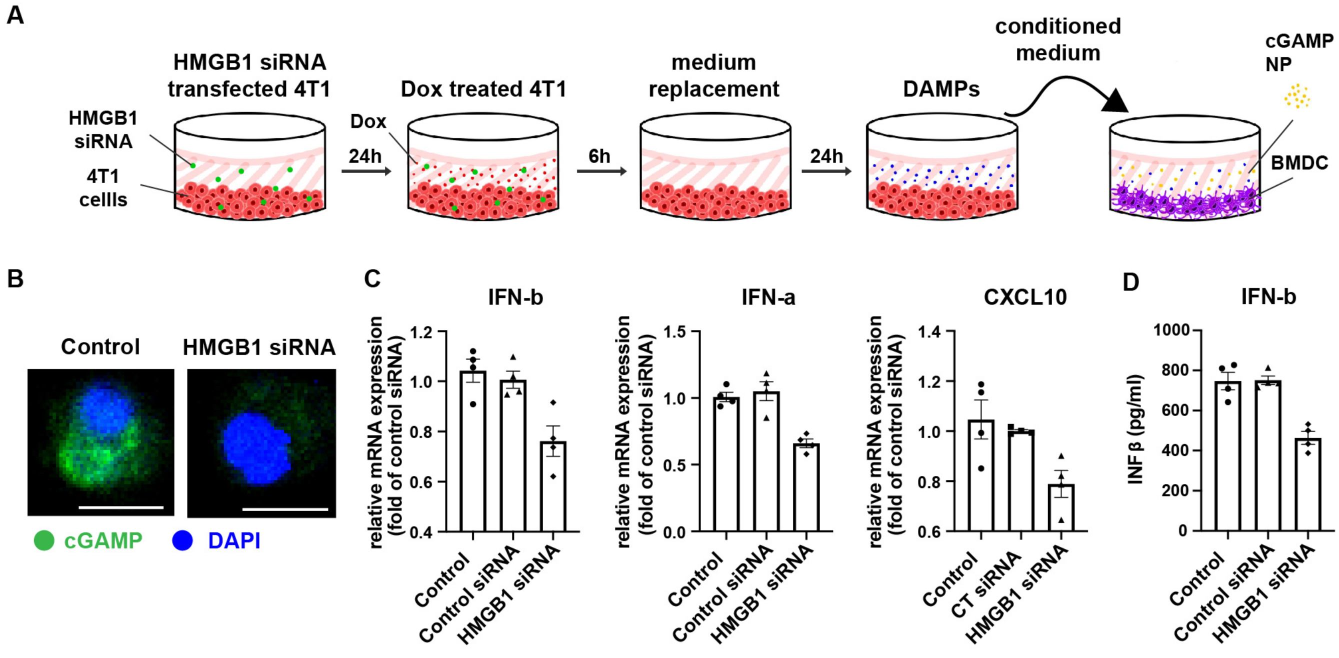

Figure S4. Viability of BMDCs and 4T1 cells after 24-hour exposure to blank or Dox@Gel. BMDCs and 4T1 cells were incubated with gels in a transwell system. Post-incubation, cell viability was assessed via MTT assay (n=3). All data are shown as the mean ± SEM. ****P<0.0001.

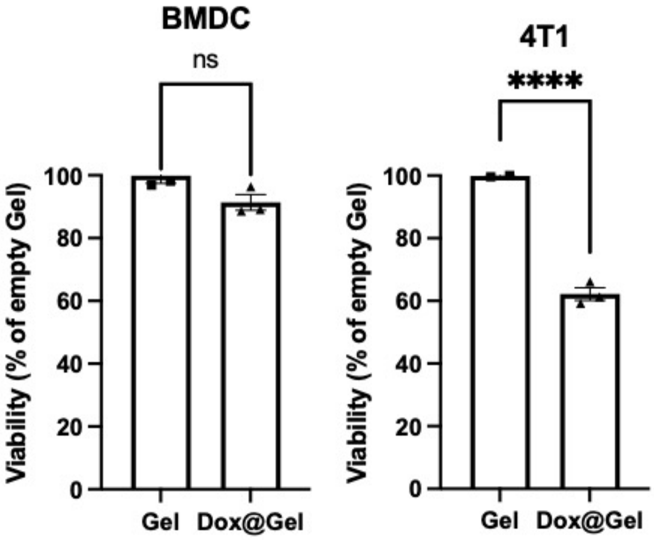

Figure S5. PD-L1 expression in 4T1 TNBC cells and immune profiling in tumor-bearing mice. (A) Flow cytometry analysis shows a significant elevation in PD-L1 expression on 4T1 cancer cells. **(B)** Immune profiling of mice implanted with orthotopic 4T1 TNBC cells reveals an increase in PD-1+ and OX40+ CD8+ T cells within both tumor-draining lymph nodes and the primary metastatic site (lung) compared to inguinal lymph nodes and lungs in healthy mice (n=5). Data are presented as mean ± SEM. *P < 0.01. **P<0.001.

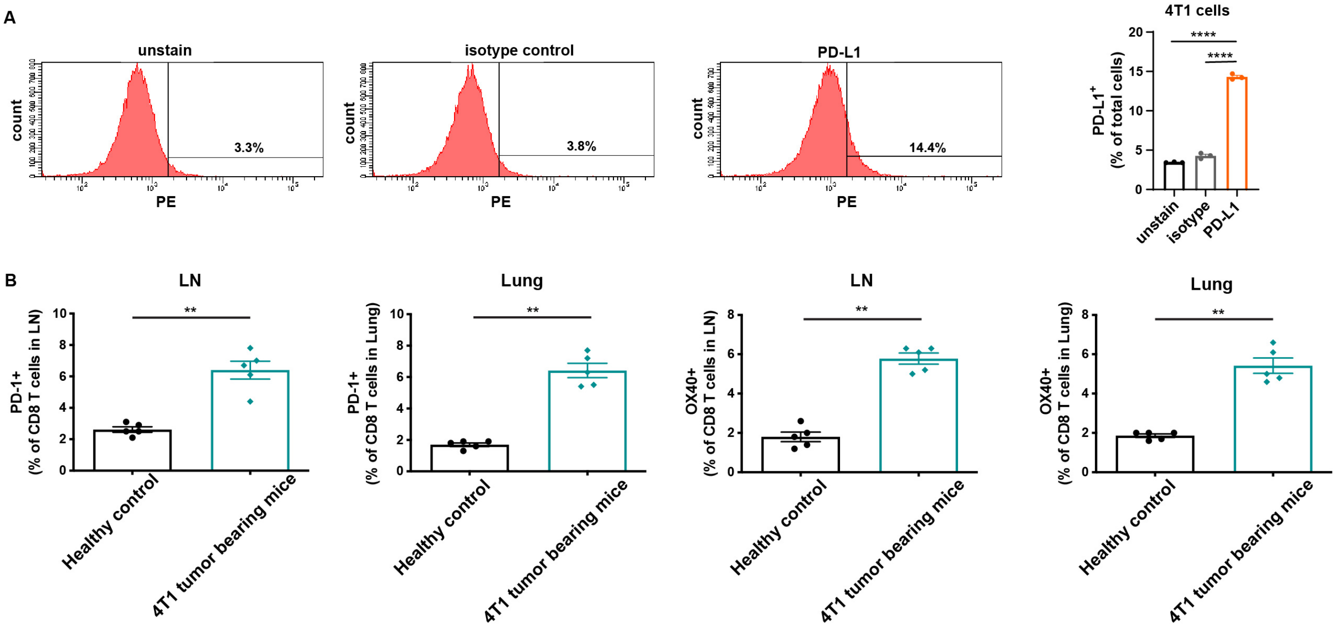

Figure S6: Gating strategy for flow cytometry to determine T cell (A) and DC subsets (B) in lymph nodes.

A

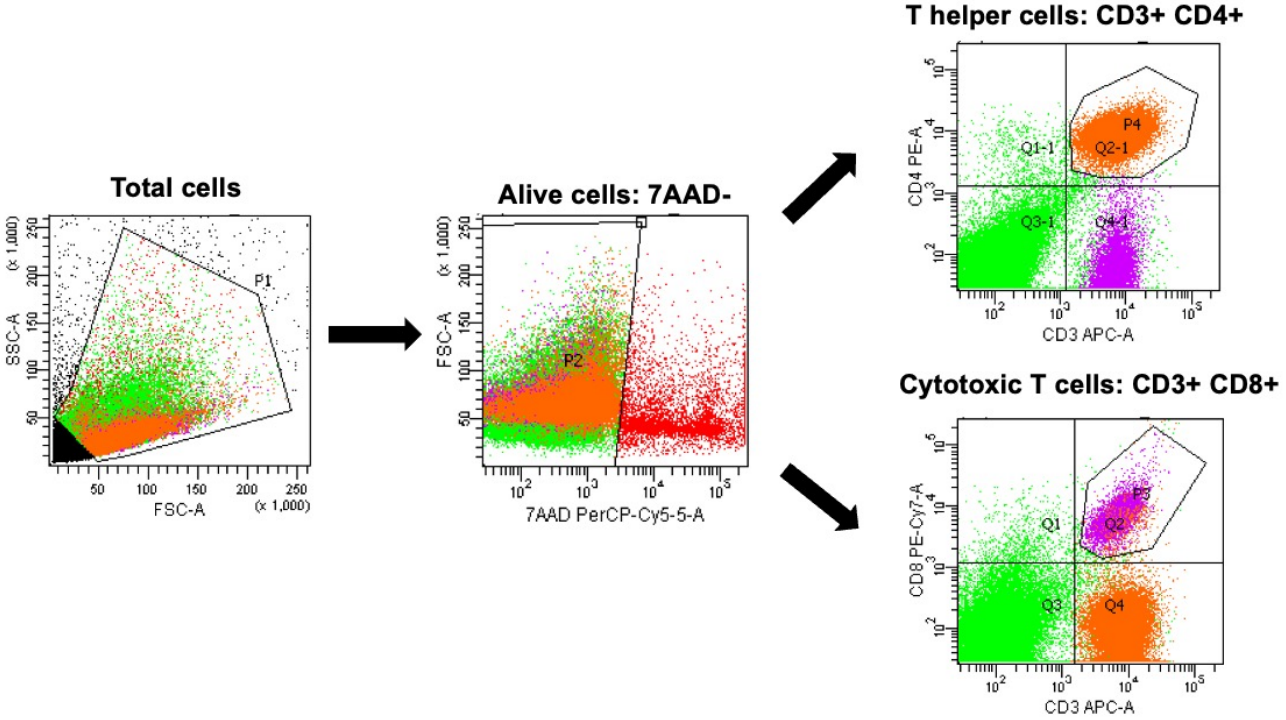

B

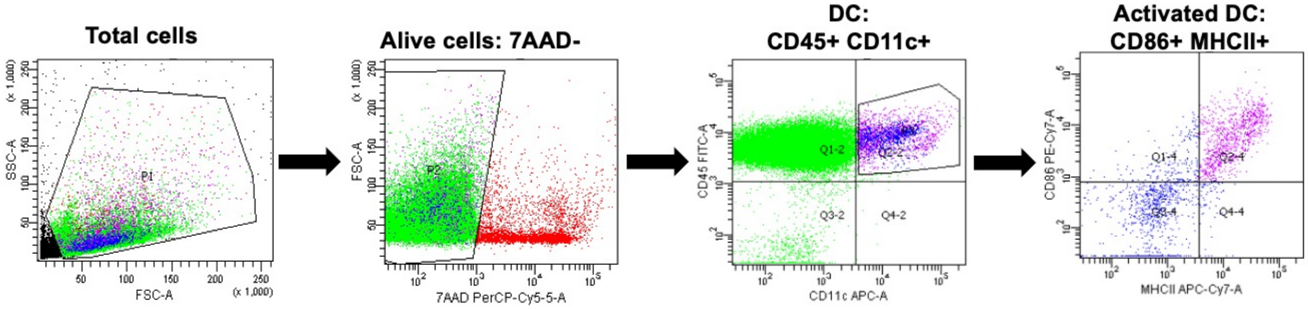

Reference:

1. Subramanian, A.; Tamayo, P.; Mootha, V. K.; Mukherjee, S.; Ebert, B. L.; Gillette, M. A.; Paulovich, A.; Pomeroy, S. L.; Golub, T. R.; Lander, E. S.; Mesirov, J. P., Gene set enrichment analysis: a knowledge-based approach for interpreting genome-wide expression profiles. *Proc Natl Acad Sci U S A* **2005,** *102* (43), 15545-50.
2. Bu, D.; Luo, H.; Huo, P.; Wang, Z.; Zhang, S.; He, Z.; Wu, Y.; Zhao, L.; Liu, J.; Guo, J.; Fang, S.; Cao, W.; Yi, L.; Zhao, Y.; Kong, L., KOBAS-i: intelligent prioritization and exploratory visualization of biological functions for gene enrichment analysis. *Nucleic Acids Res* **2021,** *49* (W1), W317-W325.
3. Whatcott, C. J.; Diep, C. H.; Jiang, P.; Watanabe, A.; LoBello, J.; Sima, C.; Hostetter, G.; Shepard, H. M.; Von Hoff, D. D.; Han, H., Desmoplasia in Primary Tumors and Metastatic Lesions of Pancreatic Cancer. *Clinical cancer research : an official journal of the American Association for Cancer Research* **2015,** *21* (15), 3561-8.
4. Frankish, A.; Diekhans, M.; Ferreira, A. M.; Johnson, R.; Jungreis, I.; Loveland, J.; Mudge, J. M.; Sisu, C.; Wright, J.; Armstrong, J.; Barnes, I.; Berry, A.; Bignell, A.; Carbonell Sala, S.; Chrast, J.; Cunningham, F.; Di Domenico, T.; Donaldson, S.; Fiddes, I. T.; Garcia Giron, C.; Gonzalez, J. M.; Grego, T.; Hardy, M.; Hourlier, T.; Hunt, T.; Izuogu, O. G.; Lagarde, J.; Martin, F. J.; Martinez, L.; Mohanan, S.; Muir, P.; Navarro, F. C. P.; Parker, A.; Pei, B.; Pozo, F.; Ruffier, M.; Schmitt, B. M.; Stapleton, E.; Suner, M. M.; Sycheva, I.; Uszczynska-Ratajczak, B.; Xu, J.; Yates, A.; Zerbino, D.; Zhang, Y.; Aken, B.; Choudhary, J. S.; Gerstein, M.; Guigo, R.; Hubbard, T. J. P.; Kellis, M.; Paten, B.; Reymond, A.; Tress, M. L.; Flicek, P., GENCODE reference annotation for the human and mouse genomes. *Nucleic Acids Res* **2019,** *47* (D1), D766-D773.
5. Li, B.; Dewey, C. N., RSEM: accurate transcript quantification from RNA-Seq data with or without a reference genome. *BMC Bioinformatics* **2011,** *12*, 323.
6. Love, M. I.; Huber, W.; Anders, S., Moderated estimation of fold change and dispersion for RNA-seq data with DESeq2. *Genome Biol* **2014,** *15* (12), 550.
7. Wu, T.; Hu, E.; Xu, S.; Chen, M.; Guo, P.; Dai, Z.; Feng, T.; Zhou, L.; Tang, W.; Zhan, L.; Fu, X.; Liu, S.; Bo, X.; Yu, G., clusterProfiler 4.0: A universal enrichment tool for interpreting omics data. *Innovation (Camb)* **2021,** *2* (3), 100141.
8. The Gene Ontology, C., The Gene Ontology Resource: 20 years and still GOing strong. *Nucleic Acids Res* **2019,** *47* (D1), D330-D338.
9. Mootha, V. K.; Lindgren, C. M.; Eriksson, K. F.; Subramanian, A.; Sihag, S.; Lehar, J.; Puigserver, P.; Carlsson, E.; Ridderstrale, M.; Laurila, E.; Houstis, N.; Daly, M. J.; Patterson, N.; Mesirov, J. P.; Golub, T. R.; Tamayo, P.; Spiegelman, B.; Lander, E. S.; Hirschhorn, J. N.; Altshuler, D.; Groop, L. C., PGC-1alpha-responsive genes involved in oxidative phosphorylation are coordinately downregulated in human diabetes. *Nat Genet* **2003,** *34* (3), 267-73.

10. Gu, Z.; Eils, R.; Schlesner, M., Complex heatmaps reveal patterns and correlations in multidimensional genomic data. *Bioinformatics* **2016,** *32* (18), 2847-9.
11. Wickham, H., *ggplot2: Elegant Graphics for Data Analysis.* Springer-Verlag New York: 2016.
12. Duarte, D.; Vale, N., Evaluation of synergism in drug combinations and reference models for future orientations in oncology. *Current Research in Pharmacology and Drug Discovery* **2022,** *3*, 100110.
